# Correction: Behavioral and psychosocial factors of quality of life among adult people living with HIV on Highly Active Antiretroviral Therapy, in public hospitals of Southwest Ethiopia

**DOI:** 10.1371/journal.pgph.0003396

**Published:** 2024-06-14

**Authors:** Abreha Addis Gesese, Yitages Getachew Desta, Endale Zenebe Behire

There is an error in reference 47. The correct reference is: Algaralleh A, Altwalbeh D, Al-Tarawneh F. Health-Related Quality of Life among Persons Living with HIV/AIDS in Jordan: An Exploratory Study. HIV/AIDS (Auckland, NZ) 2020; 12: 897.
