# Supplementary material for: Behavioral and psychosocial factors of quality of life among adult people living with HIV on Highly Active Antiretroviral Therapy, in public hospitals of Southwest Ethiopia
Source: PLOS Glob Public Health. 2022 Aug 12;2(8):e0000822. doi: 10.1371/journal.pgph.0000822 (PMC10022360; doi:10.1371/journal.pgph.0000822)
Supplement: S1 File — (DOCX) [file pgph.0000822.s001.docx]

## Operational definitions and definition of terms

For the purpose of this study, quality of life is defined as personal evaluation of how things have been going for one self, and as how the individual‘s wellbeing may be impacted over time by a disease, a disability, or a disorder.

**Overall Quality of Life**

Measured by using WHOQOL-HIV BIEF questionnaire. The percentages mean score was calculated for each participant. Then, the mean score of percent mean scores was used as a cut-off to categorize the participants as poor (less than the mean score) and good (greater than or equal to the mean score) [15, 22]

**Stigma:** is a perceived negative attribute that cause someone to devalue or think less of the whole person which is measured using mean as a cut of point. Stigmatized those with higher score value above mean and not stigmatized for below mean value[37].
**Depression** = depression is a mental disorder representing with loss of pleasure or interest, feelings of low self-esteem or guilty, depressed mood, disturbed appetite or sleep, lack of concentration or low energy. Depression was measured using Beck depression inventory (BDI-13score) by PCA considering its internal consistency and reliability with a (Crombach‘s Alpha 0.935) which is above o.7. It was measured using ordered scale from zero to three where the lower indicates minimal and the higher value indicated severe depression[38].

**Wealth quintiles:** Wealth index is a composite measure of the cumulative living standard of households and was calculated using easy-to collect data on ownership of household assets. Wealth index was generated by using statistical procedure called principal components analysis (PCA) and was categorized in to three quintiles ranked from 1^st^ quintile (poorest), 2^nd^ quintile (middle), 3^rd^ quintile (wealthiest), taken from previously used Ethiopian demographic Health Survey 2016[39].

**Satisfaction from the overall support** was measured as dissatisfied, somehow satisfied and very satisfied [22].

**Presence of psychosocial support** was measured using yes no response for each category [22].
