# Supplementary material for: Behavioral and psychosocial factors of quality of life among adult people living with HIV on Highly Active Antiretroviral Therapy, in public hospitals of Southwest Ethiopia
Source: PLOS Glob Public Health. 2022 Aug 12;2(8):e0000822. doi: 10.1371/journal.pgph.0000822 (PMC10022360; doi:10.1371/journal.pgph.0000822)
Supplement: S1 Questionnaire — (DOCX) [file pgph.0000822.s002.docx]

S1 Questionnaire. **English version questionnaire**
(DOCX)

Jimma University institute of Health School of Graduate Studies department of Population and Family Health
A. A questionnaire on Behavioral and psychosocial factors of Quality of life among Adult people living with HIV on Highly Active Antiretroviral Therapy, in public hospitals of South West Ethiopia.

**Informed Consent Form for Quantitative Part**

Good morning /afternoon? My name is………………………………… First of all, I would like to thank you for giving your time. I am working as data collector with the research team of Jimma University Institute of Health conducting research on Behavioral and psychosocial factors of Quality of life among Adult people living with HIV on Highly Active Antiretroviral Therapy, in public hospitals of South West Ethiopia. The aim of the research is to identify Behavioral and psychosocial factors of quality of life among adults PLWHIV which help for appropriate design of interventions. Communicated ideas and health statuses will be kept secret. The interview will take approximately 30 min. Are you willing to participate? Yes No If say yes, say thank you and proceed to the consent form. If say no, say thank you, do not force or reinforce to participate in the study.

**Consent form**

After the aim and objective of the study is clear, I am willing to participate in this study. Signature of participant/fingerprint……………

Date of interview………………………

Name of hospital----------------------------

Data collectors name and signature------------

1. **Screening question used during survey.**
   **Questions for assessment of self-rate quality of life and health satisfaction**
   ***Instructions.*** This assessment asks how you feel about your quality of life, health, or other areas of your life in the **past two weeks**. **Please answer all the questions.** If you are unsure about which response to give to a question, **please choose the one** that appears most appropriate. This can often be your first response. It will last for about seven minutes. Please keep in mind your standards, hopes, pleasures and concerns.

Name of hospital----------------------------

Data collectors name and signature-----------

Result of interview

1. Completed 2. Partially -completed. 3. Refuse

Q701. How would you rate your quality of life?

| **Please circle the number** | | | | |
| --- | --- | --- | --- | --- |
| very poor | Poor | Neither poor nor good | Good | Very good |
| 1 | 2 | 3 | 4 | 5 |

Q702. How satisfied are you with your health?

| Very dissatisfied | Dissatisfied | Neither dissatisfied nor satisfied | Satisfied | Very satisfied |
| --- | --- | --- | --- | --- |
| 1 | 2 | 3 | 4 | 5 |

**A - Physical domain related questioner**

Q703. To what extent do you feel that physical pain prevents you from doing what you need to do?

| Not at all | A little | A moderate amount | Very much | An extreme amount |
| --- | --- | --- | --- | --- |
| 1 | 2 | 3 | 4 | 5 |

Q704. Do you have enough energy for everyday life?

| Not at all | A little | Moderately | Mostly | Completely |
| --- | --- | --- | --- | --- |
| 1 | 2 | 3 | 4 | 5 |

Q705. Do you encounter HIV related symptoms recently?

| Very poor | Poor | Neither poor nor well | Well | Very well |
| --- | --- | --- | --- | --- |
| 1 | 2 | 3 | 4 | 5 |

Q706. How satisfied are you with your sleep?

|  |  |  |  |  |
| --- | --- | --- | --- | --- |
| Very dissatisfied | Dissatisfied | Neither dissatisfied nor satisfied | Satisfied | Very satisfied |
| 1 | 2 | 3 | 4 | 5 |

**B- Level of independence**

Q707. To what extent does mobility restrict your independence?

| Not at all | A little | A moderate amount | Very much | An extreme amount |
| --- | --- | --- | --- | --- |
| 1 | 2 | 3 | 4 | 5 |

Q708. How much do you need any medical treatmentto function in your daily life?

| Not at all | A little | A moderate amount | Very much | An extreme amount |
| --- | --- | --- | --- | --- |
| 1 | 2 | 3 | 4 | 5 |

Q709. How satisfied are you with your capacity for work?

| Very dissatisfied | Dissatisfied | Neither dissatisfied nor satisfied | Satisfied | Very satisfied |
| --- | --- | --- | --- | --- |
| 1 | 2 | 3 | 4 | 5 |

Q710. How satisfied are you with your ability to perform your daily living Activities?

| Very dissatisfied | Dissatisfied | Neither dissatisfied nor satisfied | Satisfied | Very satisfied |
| --- | --- | --- | --- | --- |
| 1 | 2 | 3 | 4 | 5 |

**C - Psychological domain related questioners**

Q711. How much do you enjoy life?

| Not at all | A little | A moderate amount | Very much | An extreme amount |
| --- | --- | --- | --- | --- |
| 1 | 2 | 3 | 4 | 5 |

Q712. How well are you able to concentrate on day to day activity?

| Not at all | A little | A moderate amount | Very much | An extreme amount |
| --- | --- | --- | --- | --- |
| 1 | 2 | 3 | 3 | 5 |

Q713. Are you able to accept your bodily appearance?

| Not at all | A little | A moderate amount | Very much | An extreme amount |
| --- | --- | --- | --- | --- |
| 1 | 2 | 3 | 4 | 5 |

Q714. How satisfied are you with yourself?

| Very satisfied | Dissatisfied | Neither satisfied nor dissatisfied | Satisfied | Very satisfied |
| --- | --- | --- | --- | --- |
| 1 | 2 | 3 | 4 | 5 |

Q715. How often do you have negative feelings, such as blue mood, despair, anxiety, depression?

| Never | Seldom | Quit often | Often | Often always |
| --- | --- | --- | --- | --- |
| 1 | 2 | 3 | 4 | 5 |

**D- Social domain related questioner**

Q716. How satisfied are you with your personal relationships?

| Very dissatisfied | Dissatisfied | Neither satisfied nor dissatisfied | Satisfied | Very satisfied |
| --- | --- | --- | --- | --- |
| 1 | 2 | 3 | 4 | 5 |

Q717. How satisfied are you with your sex life?

| Very dissatisfied | Dissatisfied | Neither satisfied nor dissatisfied | Satisfied | Very satisfied |
| --- | --- | --- | --- | --- |
| 1 | 2 | 3 | 4 | 5 |

Q718. How satisfied are you with the psychological support you get from your friends?

| Very dissatisfied | Dissatisfied | Neither satisfied nor dissatisfied | Satisfied | Very satisfied |
| --- | --- | --- | --- | --- |
| 1 | 2 | 3 | 4 | 5 |

Q719.How satisfied are you with your ability to support once family?

| Not at-all | A little | Moderately | Very much | Extreme amount |
| --- | --- | --- | --- | --- |
| 1 | 2 | 3 | 4 | 5 |

**E: Environmental domain related questioner**

Q720. How safe do you feel in your daily life?

| Not at all | A little | A moderate amount | Very much | An extreme amount |
| --- | --- | --- | --- | --- |
| 1 | 2 | 3 | 4 | 5 |

Q721. How healthy is your living physical environment?

| Not at all | A little | A moderate amount | Very much | An extreme amount |
| --- | --- | --- | --- | --- |
| 1 | 2 | 3 | 4 | 5 |

Q722. Have you enough money to meet your needs?

| Not at all | A little | A moderate amount | Very much | An extreme amount |
| --- | --- | --- | --- | --- |
| 1 | 2 | 3 | 4 | 5 |

Q723. How available to you is the information that you need in your day-to-day life?

| Not at all | A little | A moderate amount | Very much | An extreme amount |
| --- | --- | --- | --- | --- |
| 1 | 2 | 3 | 4 | 5 |

Q724. To what extent do you have the opportunity for leisure activities?

| Not at all | A little | A moderate amount | Very much | An extreme amount |
| --- | --- | --- | --- | --- |
| 1 | 2 | 3 | 4 | 5 |

Q725. How satisfied are you with the conditions of your living place?

| Very dissatisfied | Dissatisfied | Neither satisfied nor dissatisfied | Satisfied | Very satisfied |
| --- | --- | --- | --- | --- |
| 1 | 2 | 3 | 4 | 5 |

Q726. How satisfied are you with your access to health services?

| Very dissatisfied | Dissatisfied | Neither satisfied nor dissatisfied | Satisfied | Very satisfied |
| --- | --- | --- | --- | --- |
| 1 | 2 | 3 | 4 | 5 |

Q727. How satisfied are you with your mode of transportation?

| Very dissatisfied | Dissatisfied | Neither satisfied nor dissatisfied | Satisfied | Very satisfied |
| --- | --- | --- | --- | --- |
| 1 | 2 | 3 | 4 | 5 |

**F - Spiritual /religion/ personal beliefs**

Q728. To what extent do you feel accepted by the people you know?

| Not at all | A little | Moderately | Mostly | Completely |
| --- | --- | --- | --- | --- |
| 1 | 2 | 3 | 4 | 5 |

Q729. To what extent are you bothered by people blaming you for your HIV status?

| Not at all | A little | Moderately | Mostly | Completely |
| --- | --- | --- | --- | --- |
| 1 | 2 | 3 | 4 | 5 |

Q730. How much do you fear of the future?

| Not at all | A little | Moderately | Mostly | Completely |
| --- | --- | --- | --- | --- |
| 1 | 2 | 3 | 4 | 5 |

Q731. How much do you worry about death?

| Not at all | A little | Moderately | Mostly | Completely |
| --- | --- | --- | --- | --- |
| 1 | 2 | 3 | 4 | 5 |

Questionnaire code**- 0**controls**1**case

1. **Main questionnaire**

**Part1. Socio demographic factors**

| **Section 1 Socio- Demographic Data (please circle or write answers on the space provided)** | | | | | | | | | | | | | | | | | | | | | | | | |
| --- | --- | --- | --- | --- | --- | --- | --- | --- | --- | --- | --- | --- | --- | --- | --- | --- | --- | --- | --- | --- | --- | --- | --- | --- |
| 101 | | | Place of residence: | 1.rural | | | | | | 2.urban | | |  | | | | |  | | |  | | | |
| 102 | | | Gender/Sex | 1.Male | | | | | | 2.Female | | |  | | | | |  | | |  | | | |
| 103 | | | How old are you? ______ (Age in years) | | | | | | | | | |  | | | | |  | | |  | | | |
| 104 | | | Current Height in meter ________ | | | | | | | | | |  | | | | |  | | |  | | | |
| 105 | | | What is your marital status? | 1.Single | | | | | | 2.Married | | | 3.Separated | | | | | 4.Divorced | | | 5.Widowed | | | |
| 106 | | | What is your Religion | 1.Orthodox | | | | | | 2.Muslim | | | 3.Protestant | | | | | 4.Catholic | | | 5.Others specify_______ | | | |
| 107 | | | Ethnicity | 1.Oromo | | | | | | 2. Kaffa | | | 3. Dawro | | | | | 4. Amhara  5. Gurage | | | 6.Tigre  7.other(specify) | | | |
| **Section 2 Socioeconomic and Household relative Wealth index data (please circle or write answers on the space provided)** | | | | | | | | | | | | | | | | | | | | | | | | |
| 201 | What is the highest grade you attained? | | | | | | | | | | ________________________________ | | | | | | | | | | | | |  |
| 202 | Employment status | | | | 1.Government employee  2. Private business | | | | | | 3. Retired  4.Unemployed | | | | 5. Student 6. Farmer | | | | | 7. NGO employee  8. Daily laborer | | | | 9. House wife  10.Merchant |
| 203 | What is the source of income in the past 12 months | | | | 1.permanent job  2.sale of any goods other than agricultural product | | | | | | 3. Provision of any services (renting houses, car, land, livestock, equipment… | | | | 4.agricultural product  5.livestock breeding | | | | | 6.pension  7.money from government | | | | 8.non-government  9. other |
| 204 | Source of drinking water | | | | 1.pipe water  2. protected well | | | | | | 3. unprotected well | | | | 4. rain water | | | | | 5. spring | | | | 6.other |
| 205 | Does this HH own farmland? | | | | | | | | | | | | | 1=Yes | | | | | 0= No | | | | | |
| 206 | Does this HH own living house? | | | | | | | | | | | | | 1=Yes | | | | | 0= No | | | | | |
| 207 | Do you have a bank or microfinance saving account? | | | | | | | | | | | | | 1=YES | | | | | 0=NO | | | | | |
| 208 | Do you produce agricultural products? | | | | | | | | | | | | | 1=YES | | | | | 0=NO | | | | | |
| Tell me, please, if your home has the following: [INTERVIEWER: CIRCLE ALL THAT APPLY] | | | | | | | | | | | | | | | | | | | | | | | | |
| 209 | Electricity | | | | | | 1=Yes | | | | | | | | | | | | 0= No | | | | | |
| 210 | Radio | | | | | | 1=Yes | | | | | | | | | | | | 0= No | | | | | |
| 211 | Television | | | | | | 1=Yes | | | | | | | | | | | | 0= No | | | | | |
| 212 | Mobile | | | | | | 1=Yes | | | | | | | | | | | | 0= No | | | | | |
| 213 | Refrigerator | | | | | | 1=Yes | | | | | | | | | | | | 0= No | | | | | |
| 214 | Watch/clock | | | | | | 1=Yes | | | | | | | | | | | | 0= No | | | | | |
| 215 | Electric Mitad | | | | | | 1=Yes | | | | | | | | | | | | 0= No | | | | | |
| 216 | A bed with mattress (cotton/ Sponge/ Spring) | | | | | | | | | | | | | 1=Yes | | | | | 0= No | | | | | |
| What type of fuel does your household mainly use for cooking? [INTERVIEWER: ALLOW MULTIPLE ANSWERS] | | | | | | | | | | | | | | | | | | | | | | | | |
| 217 | Electricity | | | | | | 1=Yes | | | | | | | | | | | | 0= No | | | | | |
| 218 | Kerosene | | | | | | 1=Yes | | | | | | | | | | | | 0= No | | | | | |
| 219 | wood | | | | | | 1=Yes | | | | | | | | | | | | 0= No | | | | | |
| 220 | Charcoal | | | | | | 1=Yes | | | | | | | | | | | | 0= No | | | | | |
| Do you own any of the following animals? If yes, please tell me the total number of this animal you have | | | | | | | | | | | | | | | | | | | | | | | | |
| 221 | Cows/Oxen /bulls | | | | | | 1=Yes: how many________ | | | | | | | | | | | | 0= No | | | | | |
| 222 | Horses/donkey/mules | | | | | | 1=Yes: how many________ | | | | | | | | | | | | 0= No | | | | | |
| 223 | Goats | | | | | | 1=Yes: how many________ | | | | | | | | | | | | 0= No | | | | | |
| 224 | Sheep | | | | | | 1=Yes: how many_______ | | | | | | | | | | | | 0= No | | | | | |
| 225 | Chicken | | | | | | 1=Yes: how many_______ | | | | | | | | | | | | 0= No | | | | | |
| 226 | Beehives | | | | | | 1=Yes: how many_______ | | | | | | | | | | | | 0= No | | | | | |
| **Section 3-Substance use Factors (please circle or write answers on the space provided)** | | | | | | | | | | | | | | | | | | | | | | | | |
| 301 | | | In your life, have you ever used substances? | | | | | | | | | 1. Yes | | | | 1. No | | | | | | | If no skip to Q401 | |
| 302 | | | If yes to Q 301, which of the following substances have you ever used? | | | | | | | | | 1. Tobacco products (cigarettes, chewing tobacco, cigars, etc.) | | | | 2 .Alcoholic beverages (beer, wine, spirits, etc.) | | | | | | | 3. Khat chewing | |
| 303 | | | If yes to Q 301, in the past three months, how often have you used the substances you mentioned | | | | | | | | | | | | | | | | | | | | | |
| a. | | | Tobacco products (cigarettes, chewing tobacco, cigars, etc.) | | | 0.Never | | | 2.once or twice | | | 3.monthly | | | | 4.weekly | | | | | | | 6.daily or almost daily | |
| b. | | | Alcoholic beverages (beer, wine, spirits, homebrew etc.) | | | 0.Never | | | 2.once or twice | | | 3.monthly | | | | 4.weekly | | | | | | | 6.daily or almost daily | |
| C | | | khat | | | 0.Never | | | 2.once or twice | | | 3.monthly | | | | 4.weekly | | | | | | | 6.daily or almost daily | |
| **Section 4_Psychosocial support (please circle or write answers on the space provided)** | | | | | | | | | | | | | | | | | | | | | | | | |
| 401 | | Did you get (receive) support? | | | | | |  | | | | | | | | | 1. Yes | | | | | 0. No | | |
| 402 | | What kind of support did you get? (Multiple responses possible) | | | | | | 1.Emotional/Psychological  2. Financial support | | | | | | | | | 3. Physical care and support | | | | | 4. Others (specify) | | |
| 403 | | From where do you get support? (Multiple réponses are possible) | | | | | | 1= Friends 2= NGO  3=Community based organization  4=Religious based organization | | | | | | | | | 5=Government organization  6= Workplace programs | | | | | 7=family  8=Other (Specify) | | |
| 404 | | In general how satisfied are you with these support | | | | | | 1. Dissatisfied 2. Somewhat satisfied 3. Very satisfied | | | | | | | | |  | | | | |  | | |
| 405 | | Is there Someone to help you if you were confined to bed | | | | | | | | | | | | | | | 1. Yes | | | | | 1. No | | |
| 406 | | Is there Someone to give you good advice about a crisis | | | | | | | | | | | | | | | 1. Yes | | | | | 1. No | | |
| 407 | | Is there Someone to take you to the doctor if you needed it | | | | | | | | | | | | | | | 1. Yes | | | | | 1. No | | |

| **Section 5 Stigma related questions** | | | | | |
| --- | --- | --- | --- | --- | --- |
|  | **Experiences in the past 12 months** | **Never** | **Once** | **A few times** | **Often** |
| 501. | Excluded from social gatherings or activities |  |  |  |  |
| 502. | Excluded from religious activities places of worship |  |  |  |  |
| 503. | Excluded from family activities |  |  |  |  |
| 504. | Aware of being gossiped about |  |  |  |  |
| 505. | Verbally insulted harassed and/or threatened |  |  |  |  |
| 506. | Physically harassed |  |  |  |  |
| 507. | Physically assaulted |  |  |  |  |

| Depression related factors | | | |  | |
| --- | --- | --- | --- | --- | --- |
| 508d. | | Which one holds for you? Sadness | 1. I don’t feel 2. I feel sad 3. All the time/I can’t snap it 4. Am so sad/I can’t stand it |  | |
| 509d. | | Which one holds for you? Pessimism | 1. Not discouraged about the future 2. I feel discouraged 3. have nothing to look forward to 4. I feel the future is hopeless |  | |
| 510d. | | Which one holds for you? Sense of failure? | 1. I do not feel like a failure 2. I feel I have failed more than the average person 3. all I can see is as lot of failures 4. I feel I am a complete failure as a person (Parent. Husband, wife) |  | |
| 511d. | | Which one holds for you? Dislike of self | 1. I don't feel disappointed in myself   1. I am disappointed in myself  2. I am disgusted with myself  3. I hate myself |  | |
| 512d | | Which one holds for you? Insomnia | 1. I can sleep as well as usual   1. I wake up more tired in the morning than I used to  2. I wake up 1-2 hours earlier than usual and find it hard to get back to sleep  3. I wake up early every day and can't get more than 5 hours sleep |  | |
| 513d. | | Which one holds for you? Social withdrawal? | 0. I have not lost interest in other people  1. I am less interested in other people now than I used to be  2. I have lost most of my interest in other people and have little feeling for them  3. I have lost all my interest in other people and don't care about them at all |  | |
