# Supplementary material for: Behavioral and psychosocial factors of quality of life among adult people living with HIV on Highly Active Antiretroviral Therapy, in public hospitals of Southwest Ethiopia
Source: PLOS Glob Public Health. 2022 Aug 12;2(8):e0000822. doi: 10.1371/journal.pgph.0000822 (PMC10022360; doi:10.1371/journal.pgph.0000822)
Supplement: S2 Questionnaire — (DOCX) [file pgph.0000822.s003.docx]

## S2 Questionnaire. Afan Oromo version questionnaire (Gaaffanno afaan oromoo)

(DOCX)

Akkam bulte/tan, maqaan koo-------------------------jedhama. Kaniin dhufe university jimma kutaa barumsa fayyaa hawaasaa irraayu. Kaniin dhufe uummata vaayirasiin HIV dhiiga isaanii kessatti argamuun fi qoricha farra HIV fudhachaa jiraniin walqabatee gama sadarkaa qulqullina haala jireenyaa amalootaa dhibaa irrati geesisani jedhu irratti qorannaa gaggefamaa jiruuf odeeffannoo/ ragaa sassaabuudhaaf. Kanaafuu odeeffanoo tokko tokko kennuf eyyama naa kennuu dandessaa?

Deebii naa keennitu irratti maqaan kee hincaqasamu, iciitiidhaaniis kan eegamu ta’a. gaaffannon kun daqiiqaa sodomma ol nujalaa hinfuudhatu. Deebiin nuu kenitu uummata vaayirasiin dhiiga isaanii kessatti argamuu gama jijjirraa fudhanna qoricha farra HIV sadarkaa tokkoffaa irraa gara sadarkaa lammaffaa gaggefamuu irraatti sagantaawwaan hojjatamaa jiran adda addaa foyyessuuf, akkasumas tarkaanfii barbaachisaa ta’e fudhachuf gargaara. Kanaafuu, gaaffanoo kanaaf amanummadhaan deebii sirrii fi dhugaa ta’e akka naa kennitu kabajaanan si gaaffadha.

Hirmaachudhaaf waliigaleera

Hirmaachudhaaf walii hingalle

Guca Walii galtee

Waa’ee qorannichaa ibsa gabaabaa naa godhameen kaayyoo isaa sirritti hubadheera. Iciitiin koo dhuunfaa akka najalaa hintuqamne baree waliigaltee koo mallattoo kootiin raggasiisa.

Mallatoo-------------------------- Guyyaa-------------------------

**Kutaa 7ffaa: *Gaafanno quulquullina jireenyaafii sadarkaa gammachuu fayyaan walqabate.***

***Gaaffannon armaan gadii waa’ee qulqulina jireenyaa, fayyaa, yookan wantoota biraa keetti maal akka sitti dhagahaamu gaafata. Yoo deebii keennitutti shakkii qabaatte, maaloo deebii baay’ee sirri sitti fakkate filladhu. Gaaffannoon kana yeroo calqabaaf ta’uu danda’a kan deebiiftu. Kanaafuu, Sadarkaa abdii, gammachu keetiifi, dhimma jireenya keeti yaadadhu/ qalbiitti qabadhu.***

Q701. Qulqullina haala jireenya keetii( dinagdee, haawaasummaa fi fayyaa keeti) sadarkaa kamiin ibsita?

| **Maaloo deebiikee lakkofsatti marsi** | | | | |
| --- | --- | --- | --- | --- |
| Baay’ee gadi bu’aa | Gadii bu’aa | Giddugalessa | Gaarii | Baay’ee gaarii |
| 1 | 2 | 3 | 4 | 5 |

Q702.Fayyaa keettitti haagam gammadeera/ garaa sigaheera?

| Sirumayyu hingamadnee | Hingamadne | Giddugaleessa | Gamadeera | Baay’ee gamadeera |
| --- | --- | --- | --- | --- |
| 1 | 2 | 3 | 4 | 5 |

**A - Gaaffanno qaamaatiin walqabatan**

Q703. Dhukkubnii sitti dhagahaamu hojii dalaguu barbaadde irraa hagam siihaambisaa/ dhoorkaa jira?

| Siruumayyu nandhoorkine | Xiqqoonadhoorkeera | Giddugalessaatti nadhorkeera | Baay’ee nadhorkeera | Baay’ee guddaa nadhorkeera |
| --- | --- | --- | --- | --- |
| 1 | 2 | 3 | 4 | 5 |

Q704. Jiruuf jireenya guyyaatiif Humna gahaa ta’e qabdaa?

| Siruumayyu hinqabu | Xiqqoon qaba | Giddugalessatti nanqaba | Baay’een qaba | Sirriittan qaba |
| --- | --- | --- | --- | --- |
| 1 | 2 | 3 | 4 | 5 |

Q705. Yeroo dhihoo kessatti Mallattoolee HIV tiin walqabatan hagam sii mudateeraa

| Baay’ee gadii bu’aa | gadii bu’aa | Giddugalessa | Na muudateera | Baay’ee guddaa |
| --- | --- | --- | --- | --- |
| 1 | 2 | 3 | 4 | 5 |

Q706.Irriiba keetitti haagam gammadeera?

| Sirumayyu hingamadnee | Hingamadnee | Giddugalessatti gamadeea | Gamadeera | Baay’ee gamadeera |
| --- | --- | --- | --- | --- |
| 1 | 2 | 3 | 4 | 5 |

**B-Gaaffilee Sadarkaa of danda’uun walqabatan**

Q707. Of dandeessee deemuudhaaf hagam si dhorkee jira?

| Sirumayyu | Baay’ee xiqqoo | Giddugalessa | Guddaa | Baay’ee guddaa |
| --- | --- | --- | --- | --- |
| 1 | 2 | 3 | 4 | 5 |

Q708. Jireenya kee guyyaa gaggessuuf degarsa waldhaansa fayyaa haagam barbaada?

| Sirumayyu | Baay’ee xiqqoo | Giddugalessa | Guddaa | Baay’ee guddaa |
| --- | --- | --- | --- | --- |
| 1 | 2 | 3 | 4 | 5 |

Q709.Humna hojii hoojjachuudhaaf qabduuttii hammam gamaddeera?

| Baay’ee hingamadnee | Hingamadnee | Giddugalessa | Gamadeera | Baay’ee gamadeera |
| --- | --- | --- | --- | --- |
| 1 | 2 | 3 | 4 | 5 |

Q710. Jireenya kee guyyaa gagessuudhaaf dandeetti hojii hojjachuuudhaaf qabduutti hagam gamadeera

| Baay’ee hingamadnee | Hingamadnee | Giddugalessa | Gamadeera | Baay’ee gamadeera |
| --- | --- | --- | --- | --- |
| 1 | 2 | 3 | 4 | 5 |

**C - Gaaffilee xiinsammuu dhaan walqabatan**

Q711. Jireenya keetti hagam gamada

| Sirumayyu | Baay’ee xiqqoo | Giddugalessa | Guddaa | Baay’ee guddaa |
| --- | --- | --- | --- | --- |
| 1 | 2 | 3 | 4 | 5 |

Q712. Jireenya kee guyyaatti hagam qalbeefatta?

| Sirumayyu | Baay’ee xiqqoo | Giddugalessa | Guddaa | Baay’ee guddaa |
| --- | --- | --- | --- | --- |
| 1 | 2 | 3 | 3 | 5 |

Q713. Dhaabbii qaama keetii hagam amantee fuudhata

| Sirumayyu | Baay’ee xiqqoo | Giddugalessa | Guddaa | Baay’ee guddaa |
| --- | --- | --- | --- | --- |
| 1 | 2 | 3 | 4 | 5 |

Q714. Ofiikeetti hagam gamada?

| Baay’ee hingamadnee | Hingamadnee | Giddugalessa | Gamadeera | Baay’ee gamadeera |
| --- | --- | --- | --- | --- |
| 1 | 2 | 3 | 4 | 5 |

Q715. Cuurisnii badaa kan akka dhiphamuu, abdii kuutuu, fi mukaa’uu Haagam sitti dhagahama

| Sirumayyu | Darbee darbee | Irra deddeebi’ee | Yeroo baay’ee | Yeroo hunda |
| --- | --- | --- | --- | --- |
| 1 | 2 | 3 | 4 | 5 |

**D- Gaaffilee haawasummaan walqabatan**

Q716. Walqunamtii namoota wajjin qabdutti hagam gammada?

| Baay’ee hingamadnee | Hingamadnee | Giddugalessa | Gamadeera | Baay’ee gamadeera |
| --- | --- | --- | --- | --- |
| 1 | 2 | 3 | 4 | 5 |

Q717. Jiruu walqunamtii saalaatti hagam gammada?

| Baay’ee hingamadnee | Hingamadnee | Giddugalessa | Gamadeera | Baay’ee gamadeera |
| --- | --- | --- | --- | --- |
| 1 | 2 | 3 | 4 | 5 |

Q718. Deegarsa yaadaa hiriyoota keessanii irraa argatanitti hagam gammadu?

| Baay’ee hingamadnee | Hingamadnee | Giddugalessa | Gamadeera | Baay’ee gamadeera |
| --- | --- | --- | --- | --- |
| 1 | 2 | 3 | 4 | 5 |

Q719.Maatii kee deggaruuf dandeetti qabdutti hagam gammada

| Sirumayyu | Xiqqoo | Giddugaleessa | Guddaa | Baay’ee guddaa |
| --- | --- | --- | --- | --- |
| 1 | 2 | 3 | 4 | 5 |

**E: Gaaffilee naannoo dhaan walqabatan**

Q720. Jireenya kee guyyaatti nageenya haagamii sitti dhagaahaama?

| Sirumayyu | Xiqqoo | Giddugaleessa | Guddaa | Baay’ee guddaa |
| --- | --- | --- | --- | --- |
| 1 | 2 | 3 | 4 | 5 |

Q721. Naannoon/ bakkeen jireenya keetii hagam fayya qabeessa?

| Sirumayyu | Xiqqoo | Giddugaleessa | Guddaa | Baay’ee guddaa |
| --- | --- | --- | --- | --- |
| 1 | 2 | 3 | 4 | 5 |

Q722. Fedhii kee guutuudhaaf maallaqa gahaa ta’e qabdaa?

| Sirumayyu | Xiqqoo | Giddugaleessa | Guddaa | Baay’ee guddaa |
| --- | --- | --- | --- | --- |
| 1 | 2 | 3 | 4 | 5 |

Q723. Odeeffanoo Jireenya kee guyyaadhaa barbaaduu hagam aragatta?

| Sirumayyu | Xiqqoo | Giddugaleessa | Guddaa | Baay’ee guddaa |
| --- | --- | --- | --- | --- |
| 1 | 2 | 3 | 4 | 5 |

Q724. Wantoota si gammachiisan/ bashanansiisan yeroo boqonnaa keettiiti hoojjachuuf Carraa hagamii qabda?

| Sirumayyu | Xiqqoo | Giddugaleessa | Guddaa | Baay’ee guddaa |
| --- | --- | --- | --- | --- |
| 1 | 2 | 3 | 4 | 5 |

Q725. Haala/wantoota naannoo jireenya keettiti argamanitti hagam gammada?

| Baay’ee hingamadnee | Hingamadnee | Giddugalessa | Gamadeera | Baay’ee gamadeera |
| --- | --- | --- | --- | --- |
| 1 | 2 | 3 | 4 | 5 |

Q726. Tajaajila fayyaa argachuutti haagam gammada?

| Baay’ee hingamadnee | Hingamadnee | Giddugalessa | Gamadeera | Baay’ee gamadeera |
| --- | --- | --- | --- | --- |
| 1 | 2 | 3 | 4 | 5 |

Q727.Haala/ Goosa geejiba jiranitti hagam gammada?

| Baay’ee hingamadnee | Hingamadnee | Giddugalessa | Gamadeera | Baay’ee gamadeera |
| --- | --- | --- | --- | --- |
| 1 | 2 | 3 | 4 | 5 |

**F -Gaaffilee amantaa fi ilaalcha dhuunfaatiin walqabatan**

Q728. Namoota beektu biratti fuudhatama hagamiin qaba jette amanta?

| SirumayyuHinqabu | Xiqqoo nanqaba | Giddugalessa | Irra caalaatti | Guuttumaatti |
| --- | --- | --- | --- | --- |
| 1 | 2 | 3 | 4 | 5 |

Q729. HIV qabaachuu keetti Uummannii hagam nawaqasaa jira jette amanta?

| Sirumayyu | Xiqqoo | Giddugalessa | Irra caalaatti | Guuttumaatti |
| --- | --- | --- | --- | --- |
| 1 | 2 | 3 | 4 | 5 |

Q730.Fundura keetiif hagam sodaatta?

| Sirumayyu | Xiqqoo | Giddugalessa | Irra caalaatti | Guuttumaatti |
| --- | --- | --- | --- | --- |
| 1 | 2 | 3 | 4 | 5 |

Q731. Waa’ee du’aa hagam yaadda?

| Sirumayyu | Xiqqoo | Giddugalessa | Irra caalaatti | Guuttumaatti |
| --- | --- | --- | --- | --- |
| 1 | 2 | 3 | 4 | 5 |

**Hubbachiisa;**

1. **Odeffannoon Gaafanno Kanaan guuraman/ guutaman dhukkubsatoota umriin isaanii waggaa 15 fii isaa ol ta’aan qofaafi.**
2. **Odeeffanoon kutaa 1 hanga 4 jiran gaffillee abrahaamii fi amraachaw waliin guutaman/ sasaabaman kan ta’aan yoo ta’uu, gaaffileen kan biroo qobaatti kan guramanii dha. Kutaan waliin yookan qobaatti sasabaman kunnin hirmaattota qoranna kontrollitti ramadamaniifi qorricha farra HIV sadarkaa tokkooffaa fudhatan irraa qofa kan fuudhatamu dha.**

| **Kutaa 1ffaa: Gaaffilee haala hawaasaa (deebii isaa marsuudhaan ykn iddoo duwwaatti barressun ibsi)** | | | | | | | | | | | | | | | | | | | |
| --- | --- | --- | --- | --- | --- | --- | --- | --- | --- | --- | --- | --- | --- | --- | --- | --- | --- | --- | --- |
| 101 | Iddoo jireenya | 1.baadiyaa | | | | | 2.magaala | | |  | | | |  | | |  | | |
| 102 | Saala | 1.dhiira | | | | | 2.dubartii | | |  | | | |  | | |  | | |
| 103 | Waggaan kee meeqa? ______ ( umrii waggaadhaan) | | | | | | | | |  | | | |  | | |  | | |
| 104 | Hojjaa/ dheerina dhukkubsataa yeroo ammaa/ kan dhihoo ________ | | | | | | | | |  | | | |  | | |  | | |
| 105 | Haala gaa’ilaa? | 1.kan hinheerumiin/ hiinfuudhiin | | | | | 2.kan heerumtee/ fuudhee | | | 3.addaan kan bahaan | | | | 4.kan hiikee/ hiiktee | | | 5.kan jalaa du’e/ duute | | |
| 106 | Amantaa | 1.Orthodoksii | | | | | 2.Muslima | | | 3.Protestantii | | | | 4.Katoolokii | | | 5.kan biraa, _______ | | |
| 107 | Saba | 1.Oromoo | | | | | 2. Kafaa | | | 3. Daawroo | | | | 4. Amharaa  5. Guraagee | | | 6.Tiigree  7.kan biraa, adda baasi | | |
| **Kutaa 2ffaa: Gaaffilee haala hawaasa-dinaagdee fi qabeenya maatii (deebii isaa marsuudhaan ykn iddoo duwwaatti barressun ibsi)** | | | | | | | | | | | | | | | | | | | |
| 201 | Sadarkaa baruumsaa | | | | | | | ________________________________ | | | | | | | | | | |  |
| 202 | Haala hojii | | 1.Hoojii mootummaa  2. Hoojii dhuunfaa | | | | | 3. Luba kan bahee/ baatee  4.Hoojii kan hiin qabnee | | | | 5.Barataa/ barattuu 6.Qoonnaan bulaa/ buultuu | | | | 7.Qacaramaa/mtuu Hoojii mitmoootumma  8. Hojjataa guyyaa | | | 9. Haadha manaa  10.Daldaalaa/ Daldaaltuu |
| 203 | Maddii galii jii’oota 12 darban keessattii maal ture | | 1.Hoojii dhaabbataa  2. Gurguurtaa waantootaa qoonnaan argaman aalaa | | | | | 3. Tajaajila kamiyyuu keennun( Mana, lafa, loon, meeshaalee kireessuun) | | | | 4. Gurguurtaa Buu’aa qoonnaa  5. Horii hoorsiisuun | | | | 6. kaffaltii lubaa  7.Kalfaltii maallaqa mootummmaa | | | 8. kan mitmootummaa  9. Kan biraa |
| 204 | Maddi bishaan dhugaattii maalinni ( deebii tokkoo caala keennuun ni danda’ama) | | 1.Biishaan bombaa  2. Bishaan Biirii kan eegamee | | | | | 3. Bishaan Biirii kan hiineegamiin | | | | 4. Biishaan roobaa | | | | 5. Laga | | | 6.Kan biraa |
| 205 | Lafa qonnaa qabduu? | | | | | | | | | | 1=Eeyyee | | | | 0= Lakki | | | | |
| 206 | Mana jireenyaa qabduu? | | | | | | | | | | 1=Eeyyee | | | | 0= Lakki | | | | |
| 207 | Qusannaa baankii yookan waldaa qusannaa qabduu? | | | | | | | | | | 1=Eeyyee | | | | 0= Lakki | | | | |
| 208 | Oomisha qonnaa qabduu? | | | | | | | | | | 1=Eeyyee | | | | 0= Lakki | | | | |
| waantootaa armaan gadii kessaa mana kessaniittii maal faatu argama ( waantoota jiran hunda fillachuun nidanda’ama) | | | | | | | | | | | | | | | | | | | |
| 209 | Eletrikii | | | | 1=Eeyyee | | | | | | | | | | 0= Lakki | | | | |
| 210 | Raadiyoonii | | | | 1=Eeyyee | | | | | | | | | | 0= Lakki | | | | |
| 211 | Televizhinii | | | | 1=Eeyyee | | | | | | | | | | 0= Lakki | | | | |
| 212 | Moobaayilii | | | | 1=Eeyyee | | | | | | | | | | 0= Lakki | | | | |
| 213 | Firiijii | | | | 1=Eeyyee | | | | | | | | | | 0= Lakki | | | | |
| 214 | Sa’aatii | | | | 1=Eeyyee | | | | | | | | | | 0= Lakki | | | | |
| 215 | Eelee eleetriikii | | | | 1=Eeyyee | | | | | | | | | | 0= Lakki | | | | |
| 216 | Siree fiiraashii wajjiin ( jiirbii/ ispoonjii/ spriingii) | | | | | | | | | | 1=Eeyyee | | | | 0= Lakki | | | | |
| Mana keessan kessatti nyaata qoophessuuf maal fayadamtuu ( Waantoota fayyadamtan hunda fillachuun niidanda’ama) | | | | | | | | | | | | | | | | | | | |
| 217 | Eeletriki | | | | 1=Eeyyee | | | | | | | | | | 0= Lakki | | | | |
| 218 | Gaazii aadii | | | | 1=Eeyyee | | | | | | | | | | 0= Lakki | | | | |
| 219 | Qoraan | | | | 1=Eeyyee | | | | | | | | | | 0= Lakki | | | | |
| 220 | Kasala | | | | 1=Eeyyee | | | | | | | | | | 0= Lakki | | | | |
| Kan armaan gadii kessaa horiiwwaan isaan kam qabduu ( kan qabdan hunda fillachu nidandessuu) waantoota jiran hunda fillachuun nidanda’ama | | | | | | | | | | | | | | | | | | | |
| 221 | Loon | | | | 1=Eeyyee: meeqa________ | | | | | | | | | | 0= Lakki | | | | |
| 222 | haarree/ farda/ gaangee | | | | 1=Eeyyee: meeqa | | | | | | | | | | 0= Lakki | | | | |
| 223 | Ree’ee | | | | 1=Eeyyee: meeqa | | | | | | | | | | 0= Lakki | | | | |
| 224 | Hoolaa | | | | 1=Eeyyee: meeqa | | | | | | | | | | 0= Lakki | | | | |
| 225 | Hindaanqoo | | | | 1=Eeyyee: meeqa | | | | | | | | | | 0= Lakki | | | | |
| 226 | Gaagura dammaa | | | | 1=Eeyyee: meeqa | | | | | | | | | | 0= Lakki | | | | |
| **Kutaa3- Gaaffilee amalaan/ Araadaan walqabatan (deebii isa marsuudhaan ykn iddoo duwwaatti barressun ibsi)** | | | | | | | | | | | | | | | | | | | |
| 301 | Umrii keessan kessatti, dhuugaatii alkooliiqabu, tamboo, yookan immoo caatii fayyadamtanii beektuu? | | | | | | | | 1. Eeyyee | | | | 1. Lakkii | | | | | Lakkii yoo ta’e gara Gaafii 401 tti darbi | |
| 302 | Gaffii 301 dhaaf deebiin keessan Eeyyee yoo ta’e , kan armaan gadii kessaa isa kam faayyadamtanii beektuu ? | | | | | | | | 1.Tamboo (xuuxuu, qama’uu) | | | | 2 .Dhuugaatii alkoolii (biiraa, wayinii, farsoo, araqee, daadhii ) | | | | | 3. caatii qama’uu | |
| 303 | Waantoota nan fudhadha jettan , ji’a sadii darban keessattii yeroo haamamiif fayadamtan | | | | | | | | | | | | | | | | | | |
| a. | Tamboo (xuuxuu, qama’uu) | | | 0.sirumayyuu | | 2. yeroo tokko yookan lama | | | 3.Ji’atti yeroo tokko | | | | 4.torbee torbeetti | | | | | 6. guyyaa guyyaatti/ guyyaa guyyaa dhaan jechuun ni danda’ama | |
| b. | Dhuugaatii alkoolii  ( biiraa, wayinii, farsoo, araqee, daadhii) homebrew etc.) | | | 0.sirumayyuu | | 2. yeroo tokko yookan lama | | | 3. Ji’atti yeroo tokko | | | | 4. torbee torbeetti | | | | | 6. guyyaa guyyaatti/ guyyaa guyyaa dhaan jechuun ni danda’ama | |
| C | Caatii | | | 0.sirumayyuu | | 2. yeroo tokko yookan lama | | | 3. Ji’atti yeroo tokko | | | | 4. torbee torbeetti | | | | | 6. guyyaa guyyaatti/ guyyaa guyyaa dhaan jechuun ni danda’ama | |

| **Boqqonnaa afraffaa_ Gargaarsa maatii fi haawaasaa ( deebi keenammu marsuudhaan ykn iddo isaatti barreesuun ibsaa)** | | | | |
| --- | --- | --- | --- | --- |
| 401 | Gargaarsa argattee beektaa? | 1. Eeyyee | 0. lakkii | Gaaffii 401 tiif deebiin lakki yoo ta’ee, gara gaaffii 501 tti darbaa |
| 402 | Gargaarsa akkamii argattee(deebii tokko caala keennun ni danda’ama) | 1.deeggarsa xiinsammuu  2. deeggarsa maallaqaa | 3. kuunuunsa qaamaa | 4. kan biraa yoo jiraatee ibsi |
| 403 | Deeggarsa/ gargaarsa kana eenyuu irraa argata?(deebii tokko caala keennun ni danda’ama) | 1= hiiriyoota irraa 2=dhaabbilee mitmootumma irraa 3=dhaabbilee haawasummaa irraa  4=dhaabbilee amantaa irraa | 5=dhaabbilee mootummaa irraa  6= sagantaa bakka/ iddoo hojii irraa | 7=maatii irraa  8=kan biraa (ibsi) |
| 404 | Waluumaa galattii gargaarsa kanatti haamam gamadde | 1. Hin gamadnee 2. Amma tokkoo gamadeera 3. Baay’een gamade |  |  |
| 405 | Namnii sii dhuukubsachisuu jiraa | | 1. Eeyyee | 1. Lakkii |
| 406 | yeroo rakkoo namnii gorsa gaarii sii keennuu jiraa? | | 1. Eeyyee | 1. Lakkii |
| 407 | yeroo mana yaala deemuu barbaadde, namnii sigeessu jiraa? | | 1. Eeyyee | 1. Lakkii |

**Kutaa 5 ffaa:Gaffanno Hordooffi odeffanno raabsaa qoricha farra HIV ( Amraachew baqqala). Gaafannon kunnin hirmaattota qoranna kontrollitti ramadamaniifi qorricha farra HIV sadarkaa tokkooffaa fudhatan irraa qofa kan fuudhatamu dha. ( deebii kennamu marsuudhaan ykn iddoo isaatti barreesuun ibsaa)**

|  | | | | | | |
| --- | --- | --- | --- | --- | --- | --- |
| 501 | Qorannaa HIV haala kamiin taasiste/ taasiistan | | | | 1=Feedhii dhuunfaatiin (VCT) 2=kakka’umsa ogeessa fayyaatiin(PITC) 3= Haadha irra gara ilmootti akka hindarbine ittisuuf (PMTCT)  4=Qorannaa fayyaatiin 5=kan biraa, ibsi | |
| 502 | Qooricha farra HIV yeroo fudhachuu jalqabde akka HIVin dhiiga kee kessa jiru abbaa warraa/ haadha manaa ykn maatii keetti ifa gootee turtee? | | | | 1. Eeyyee | 0. Lakkii |
| 503 | Qoricha farra HIV fayyadamuu keetin dura, qorrichicha sababoota biraatiif itti fayyadamtee beektaa? | | | | 1. Eeyyee | 0. Lakkii |
| 504 | Gaaffii lakk 503 tiif deebiin eeyyee yoo ta’e, sababni itti fayyadamteef maal ture? | | 1.ulfaan walqabatee | | 2.HIV tiif wantootni saaxiiluu danda’an waan uumamaniif | 3.Adda hiin baanee/ hin beekamnee |
| 505 | yeroo qoricha farra HIV fudhachuu jalqabdetti, dhibee sombaatiin dhukkubsataa turtee | | 1. Eeyyee | | 1. Lakkii | |
| 506 | Qorricha farra HIV fudhachuu addaan kuttee/ dhabdee beektaa | | | | 1.Eeyyee | 0.Lakkii |
| 507 | Gaaffii lakk 506 tiif deebiin kee eeyyee yoo ta’e, sababnii addaan kutteef maal ture? (deebii tokko caala keennun ni danda’ama) | 1.Qorrichichi dhumuu isaatiin  2.Dhibee dhaan | | 3.Qorichicha fudhachuuf waantan nyaadhu waan hinqabneef | 4. Miidhaa qorrichaa  5.Qorichicha manatti gatee iddoo biraa deemee waanan tureef  6. Dagachuun | 7.Qorichicha iddoon lafa kaa’ee dhabuun/ baduu  8. kan biraa yoo jiraate ibsi |
| 508 | Beelama Hoordooffi dhaabbata fayyaati qabdu haaftee beektaa | | | | 1. Eeyyee | 1. Lakkii |
| 509 | Gaaffii lakk 508 tiif, deebiin eeyyee yoo ta’e sababni kee maal ture? (deebii tokko caala keennun ni danda’ama) | | 1.Dhaabatnii fayyaa fagoo waan ta’eef  2.Rakkoo geejiibaa | | 3.Hanqiina maallaqaa | 4. Dhibee dhaan  5.Kan biraa yoo jiraate ifa godhi |
