# Supplementary material for: Behavioral and psychosocial factors of quality of life among adult people living with HIV on Highly Active Antiretroviral Therapy, in public hospitals of Southwest Ethiopia
Source: PLOS Glob Public Health. 2022 Aug 12;2(8):e0000822. doi: 10.1371/journal.pgph.0000822 (PMC10022360; doi:10.1371/journal.pgph.0000822)
Supplement: S3 Questionnaire — (DOCX) [file pgph.0000822.s004.docx]

## S3 Questionnaire. Amharic version questionnaire (የአማርኛመጠይቅ)

እንደምንአደርሽ/ሩስሜ .....................................................ይባላል፡፡የመጣሁት ከጅማ ዩኒቨርሲቲ የማህበረሰብ ጤና ስነህዝብና ቤተሰብ ትምህርት ክፍል ነው፡፡አሁን ከቫይረሱ ጋር የሚኖሩ ሰዎች የህይወት ጥራትን የሚያጉዋድሉ ባህሪያዊ ና ድጋፋዊ ተያያዥ ምክንያቶች በሚል ጥናት ላይ መረጃ ለመሰብሰብ ነው የመጣሁት፡፡ ስለዚህ አንዳንድ መረጃዎችን ሊሰጡኝ ፍቃደኛ ነዎት?

በሚመለሰዉ ምላሽ ላይ ስምዎት አይጻፍም፤ሚስጥራዊነቱም የተጠበቀነው፡፡ቃለመጠይቁከሰላሳ ደቂቃበላይአይወስድብንም፡፡ምላሾቹየጸረኤችአይቪመድሀኒቶችንለማሻሻልእንዲሁምከቫይሩጋርየሚኖሩሰዎችንየሕይወትጥራትከማወቅምባለፈምክንያቶችንበመለየትአስፈላጊእርምጃዎችንለመውሰድይረዳናል፡፡ስለዚህበታማኝነትለሚጠየቁትክክለኛውንእናእውነተኛውንመልስእንዲሰጡንበትህትናእጠይቃለው፡፡

ለመሳተፍተስማምቻለው

ለመሳተፍአልተስማማሁም

**የውልቅጽ**

ስለጥናቱበአጭሩገለጻተደርጎልኝስለአላማውበትክክልተረድቻለው፡፡የግልሚስጥሬንየማይነካእንደሆነአውቄስምምነቴንበፊርማዬአረጋግጣለው፡፡

ፊርማ --------------------------

ቀን-------------------------

የመጠይቅቁጥር0በከፍተኛየታመመ1የመጀመሪያደረጃምደሃኒትላየለ/ች

የጤናተቋሙስም----------------------------

የመረጃሰብሳቢውስምእናፊርማ---------------------

- 1. **መመልመያመጠይቅ**

**ክፍል 7የህይወትጥራትደረጃመጠይቅ፡፡**

የሚከተሉትጥያቄዎችየእርሶንስሜትየሚዳስሱናቸው፡፡ከተሰጡትአማራጮችየሚስማማዎትንእንዲመርጡበአክብሮትእንጠይቆታለን፡፡

Q701.የኑሮዎትን፡መልካምነት፤ማለትም (የኢኮኖሚያዊ፡ማህበራዊናጤንነትዎን፡ሁኔታ/ጥራት) እንዴትይገልፁታል?

| የመረጡትንቁጥርያክብቡ | | | | |
| --- | --- | --- | --- | --- |
| በጣምዝቅተኛ | ዝቅተኛ | መካከለኛ | ጥሩ | በጣምጥሩ |
| 1 | 2 | 3 | 4 | 5 |

Q702. በጤንነትዎምንያህልተደስተዋል?

| በፍጹምአልተደሰትኩም | አልተደሰትኩም | መካከለኛ | ተደስቻለው | በጣምተደስቻለው |
| --- | --- | --- | --- | --- |
| 1 | 2 | 3 | 4 | 5 |

**ሀ. ዐካላዊመጠይቆች**

Q703. የሚሠማዎትህመምምንያክልመስራትየፈለጉትንእንዳያከናውኑአድርግዎታል፡፡

| በፍፁም  አልከለከለኝም | በጥቂቱከልክሎኛል | በመጠኑ | ብዙ | በጣምብዙ |
| --- | --- | --- | --- | --- |
| 1 | 2 | 3 | 4 | 5 |

Q704. ለእለትተእለትህይወትዎየሚሆንበቂሃይልአለዎት?

| በፍፁምየለኝም | በጥቂቱአለኝ | በመጠኑአለኝ | በአብዛኛውአለኝ | በሚገባአለኝ |
| --- | --- | --- | --- | --- |
| 1 | 2 | 3 | 4 | 5 |

Q705. በዚህዐጭርጊዘዉስትየህመምምልክትዐጋትሞሃል?

| በፍፁምዐላጋጠመኝም | ዐላጋጠመኝም | በመጠኑዐጋትሞኛል | ዐጋትሞኛል | በጣምዐጋትሞኛል |
| --- | --- | --- | --- | --- |
| 1 | 2 | 3 | 4 | 5 |

Q706.በእንቅልፍየሚያገኙትእርካታምንያክልነው?

|  |  |  |  |  |
| --- | --- | --- | --- | --- |
| በጣምአልረካም | አልረካም | በመጠኑእረካሁ | እረካሁ | በጣምእረካሁ |
| 1 | 2 | 3 | 4 | 5 |

**ለ. ማንነትንየማወቅደረጃመጠይቅ**

Q707. ምንያህልራስህንችለህዐለመንቀሳቀስጫናዐድርጎብሃል?

| በፍፁም | በትንሹ | በመጠኑእረካሁ | እረካሁ | በጣምእረካሁ |
| --- | --- | --- | --- | --- |
| 1 | 2 | 3 | 4 | 5 |

Q708. የእለትተእለትእንቅስቃሴዎንለማካሄድምንያክልየህክምናእርዳታያስፈልገዎታል ?

| በፍፁም | በትንሹ | በመጠኑእረካሁ | እረካሁ | በጣምእረካሁ |
| --- | --- | --- | --- | --- |
| 1 | 2 | 3 | 4 | 5 |

Q709. ስራለመስራትባሰቡጊዜየሚኖርዎአቅምምንያክልይረካሉ

| በጣምአልረካም | አልረካም | በመጠኑእረካሁ | እረካሁ | በጣምእረካሁ |
| --- | --- | --- | --- | --- |
| 1 | 2 | 3 | 4 | 5 |

Q710. የእለትተእለትተግባርዎትበማከናወንብቃትዎምንያክልይረካሉ?

| በጣምአልረካም | አልረካም | በመጠኑእረካሁ | እረካሁ | በጣምእረካሁ |
| --- | --- | --- | --- | --- |
| 1 | 2 | 3 | 4 | 5 |

**ሐ. የስነልቦናመጠይቅ**

Q711. በህይወትህ/ሽምንያክልደስተኛነህ/ሽ?

| በፍፁም | በትንሹ | በመጠኑ | በአብዛኛው | ሙሉበሙሉ |
| --- | --- | --- | --- | --- |
| 1 | 2 | 3 | 4 | 5 |

Q712. በእለትተእለትተግባሮትትኩረትየማድርግብቃትዎምንያክልነው

| በፍፁም | በትንሹ | በመጠኑ | በአብዛኛው | ሙሉበሙሉ |
| --- | --- | --- | --- | --- |
| 1 | 2 | 3 | 3 | 5 |

Q713. የሰውነትዎንአቋምአምነውይቀበላሉ ?

| በፍፁም | አልቀበልም | በመጠኑ | እቀበላለሁ | በጣምዕቀበላለሁ |
| --- | --- | --- | --- | --- |
| 1 | 2 | 3 | 4 | 5 |

Q714. በብቃትዎምንያክልይረካሉ?

| በጣምአልረካም | አልረካም | በመጠኑእረካሁ | እረካሁ | በጣምእረካሁ |
| --- | --- | --- | --- | --- |
| 1 | 2 | 3 | 4 | 5 |

Q715. ምንያክልጥሩያልሆነስሜቶችማለትምእንደተስፋመቁረጥ፣ጭንቀትእናድብርትተሠምትዎትያውቃል?

| በፍፁምአላውቅም | አልፎአልፎ | በተደጋጋሚ | ብዙጊዜ | ሁልጊዜ |
| --- | --- | --- | --- | --- |
| 1 | 2 | 3 | 4 | 5 |

**መ- ማህበራዊኑሮንየሚያትቱመጠይቆች**

Q716. ከሰዎችጋርባለውግንኙነትምንያክልይረካሉ?

| በጣምአልረካም | አልረካም | በመጠኑእረካሁ | እረካሁ | በጣምእረካሁ |
| --- | --- | --- | --- | --- |
| 1 | 2 | 3 | 4 | 5 |

Q717. በወሲብህይወትዎምንያክልእርካታንያገኛሉ ?

| በጣምአልረካም | አልረካም | በመጠኑእረካሁ | እረካሁ | በጣምእረካሁ |
| --- | --- | --- | --- | --- |
| 1 | 2 | 3 | 4 | 5 |

Q718. ከጓደኞችህበሚያገኙትየሀሳብድጋፍምንያክልይረካሉ

| በጣምአልረካም | አልረካም | በመጠኑእረካሁ | እረካሁ | በጣምእረካሁ |
| --- | --- | --- | --- | --- |
| 1 | 2 | 3 | 4 | 5 |

Q719. በተሰብህንበመርዳትህምንያህልትረካለህ?

| በፍፁም | በትንሹ | በመጠኑ | በአብዛኛው | ሙሉበሙሉ |
| --- | --- | --- | --- | --- |
| 1 | 2 | 3 | 4 | 5 |

**ሠ. አካባቢያዊመጠይቆች**

Q720. በኑሮሁኔታዎምንያክልይረካሉ

| በፍፁም | በትንሹ | በመጠኑ | በአብዛኛው | ሙሉበሙሉ |
| --- | --- | --- | --- | --- |
| 1 | 2 | 3 | 4 | 5 |

Q721. የሚኖሩበትአከባቢምንያክልጤናማነው?

| በፍፁም | በትንሹ | በመጠኑ | በአብዛኛው | ሙሉበሙሉ |
| --- | --- | --- | --- | --- |
| 1 | 2 | 3 | 4 | 5 |

Q722. የፈለጉትንአግኝተውለመኖርየሚያስችልበቂገንዘብአልዎት ?

| በፍፁም | በትንሹ | በመጠኑ | በአብዛኛው | ሙሉበሙሉ |
| --- | --- | --- | --- | --- |
| 1 | 2 | 3 | 4 | 5 |

Q723. የእለቱየሚያስፈልግዎትንመረጃየማግኘትእድልአለዎት ?

| በፍፁም | በትንሹ | በመጠኑ | በአብዛኛው | ሙሉበሙሉ |
| --- | --- | --- | --- | --- |
| 1 | 2 | 3 | 4 | 5 |

Q724. ለመዝናኛየሚሆንምንያክልጊዜአለዎት

| በፍፁም | በትንሹ | በመጠኑ | በአብዛኛው | ሙሉበሙሉ |
| --- | --- | --- | --- | --- |
| 1 | 2 | 3 | 4 | 5 |

Q725. በምትኖሩበትቦታያሉሁነታዎችምንያህልያረካዎታል?

| በጣምአልረካም | አልረካም | በመጠኑእረካሁ | እረካሁ | በጣምእረካሁ |
| --- | --- | --- | --- | --- |
| 1 | 2 | 3 | 4 | 5 |

Q726. የጤናአገልግሎትመስጫተቋማትንከማግኘትአንፃርምንያክልይረካሉ?

| በጣምአልረካም | አልረካም | በመጠኑእረካሁ | እረካሁ | በጣምእረካሁ |
| --- | --- | --- | --- | --- |
| 1 | 2 | 3 | 4 | 5 |

Q727. በመጓጓዣመንገዶችምንያክልይረካሉ

| በጣምአልረካም | አልረካም | በመጠኑእረካሁ | እረካሁ | በጣምእረካሁ |
| --- | --- | --- | --- | --- |
| 1 | 2 | 3 | 4 | 5 |

**ረ. ሀይማኖታዊ /ዕምነትተኮርመጠይቆች**

Q728. በሰዎችዘንድምንያህልተቀባይነትዐለኝብለህ/ሽታስባለህ/ሽ?

| በፍፁም | በትንሹ | በመጠኑ | በአብዛኛው | ሙሉበሙሉ |
| --- | --- | --- | --- | --- |
| 1 | 2 | 3 | 4 | 5 |

Q729. ምንያህልሰዎችከቫይረሱጋርበመኖርሽ/ህኮንነውሽ/ህያውቃሉ?

| በፍፁም | በትንሹ | በመጠኑ | በአብዛኛው | ሙሉበሙሉ |
| --- | --- | --- | --- | --- |
| 1 | 2 | 3 | 4 | 5 |

Q730. ነገን (መጪውንጊዜ) ፈርተሸ/ህታውቂያለሽ/ህ?

| በፍፁም | በትንሹ | በመጠኑ | በአብዛኛው | ሙሉበሙሉ |
| --- | --- | --- | --- | --- |
| 1 | 2 | 3 | 4 | 5 |

Q731. ምንያህልስለመሞትአሳስቦሽ/ህታውቂያለሽ/ህ?

| በፍፁም | በትንሹ | በመጠኑ | በአብዛኛው | ሙሉበሙሉ |
| --- | --- | --- | --- | --- |
| 1 | 2 | 3 | 4 | 5 |

- 1. **ዋናውመጠይቅ**

**ክፍል 1-የስነ-ሕዝብመረጃ (**መልሶቹንበማክበብወይምበባዶቦታውላይበመፃፍይግለ)

| **101** | የመኖሪያስፍራ | 1. ገጠር | 0. ከተማ |  |  |  |  |
| --- | --- | --- | --- | --- | --- | --- | --- |
| **102** | እድሜ____________ | |  |  |  |  |  |
| **103** | ጾታ | 1. ወንድ | 0. ሴት |  |  |  |  |
| **104** | የቅርብ/ አሁንየታካሚው/ዋቁመትበሜትር __________________ | | | | | | |
| **105** | የጋብቻሁኔታ | 1. ያላገባ/ች | 2. ያገባ/ች | 3. ያገባ/ችአብሮየማይኖሩ | 4. የፈታ/ች | 5. ባልየሞተባት |  |
| **106** | ሀይማኖት | 1. ሙስሊም | 2. ኦርቶዶክስ | 3. ፕሮቴስታንት | 4. ካቶሊክ | 5. ሌላ |  |
| **107** | ብሔር | 1. ኦሮሞ | 2. ከፋ | 3. ዳውሮ | 4.አማራ | 5. ትግሬ  6. ጉራጌ | 7. ሌላ |

ክፍል 2 ማህበራዊ-ኢኮኖሚያዊእና**የሀብትመጠይቅ (**መልሶቹንበመክበብወይምበባዶቦታውላይበመፃፍይግለÌ)

| 201 | የትምህርትደረጃ | __________________________________________ | | | |  |
| --- | --- | --- | --- | --- | --- | --- |
| 202 | የስራሁኔታ | 1. የመንግስትሰራተኛ  2. የNGO ተቀጣሪ  3. የቀንሰራተኛ  4. የቤትእመቤት | 5. ገበሬ  6. የግልሥራ  7. ነጋዴ | | | 8. ተማሪ  9. ሌላ …………………………….…….. |
| 203 | በባለÕት 12 ወራትዉስጥየገቢምንÃዎምንነበር? | 1.ቐሚቅጥር  2.የግብርናዉጠትበመሸጥ  3. ከመንግስትየሚገኝገንዘብ | 4. ለስራበማዋል (በትኪራይ, መኪና, መረት, የቁምከብት, አቃዎቸን,,,) | | | 5.መንግስታዊካልሆኑ  6.ከሌሎች ____________ |
| 204 | የሚጠጡትዉሀምንጩከየትነዉ?  (ከአንድበላይመልስይቻላል) | 1. የቧንቧውሃ  2. የቢሪ /የጉድጓድውሃ  3. የመሬትውሃየተከለለ | 4. የመሬትውሃያልተከለከለ  5. የዝናብዉሃ | | | 6.የወንዝ  7. የጠበልዉሀ  8. ሌላ _______ |
| 205 | የመኖሪያቤት | 1. አለኝ | 0. የለኝም | | |  |
| 206 | የእርሻመሬትአለዎት | 1. አዎ | 0. የለም | | |  |
| 207 | ከቤተሰብዎአባልውስጥየባንክአካውንትያለውአለ? | 1. አዎ | 0. የለም | | |  |
| 208 | የግብርናዉጤቶችንያመርታሉወይ? | 1.አዎ | 2. አላመርትም | | |  |
|  | **በቤትዎከሚከተሉትውስጥየትኞቹይገኛሉ (ያለውንሁሉመምረጥይቻላል)** | | | | | |
| 209 | ኤልክትሪክ | 1. አለ | 0. የለም | | |  |
| 210 | ሬዲዎ | 1. አለ | 0. የለም | | |  |
| 211 | ቴሌቪዥን | 1. አለ | 0.የለም | | |  |
| 212 | ሞባይል /ተንቀሳቃሽስልክ | 1. አለ | 0. የለም | | |  |
| 213 | ፍሪጅ/ማቀዝቀዣ | 1. አለ | 0. የለም | | |  |
| 214 | ሰዓት | 1. አለ | 0. የለም | | |  |
| 215 | የኤሌክትሪክምጣድ | 1. አለ | 0. የለም | | |  |
| 216 | አልጋናፍራሽ | 1. አለ | 0. የለም | | |  |
|  | **በቤትዎውስጥምግብለማብሰልምንአይነትማገዶይጠቀማሉ? (የሚጠቀሙትንሁሉመምረጥይቻላል)** | | | | | |
| 217 | ኤሌክትሪክ | 1. አለ | 0. የለም |  | | |
| 218 | ነጭጋዝ | 1. አለ | 0. የለም |  | | |
| 219 | እንጨት | 1. አለ | 0. የለም |  | | |
| 220 | ከሰል | 1. አለ | 0. የለም |  | | |
|  | **ከሚከተሉትውስጥየትኞቹእንስሳትአለዎት? (ያሉትንሁሉመምረጥይቻላል)** | | | | | |
| 221 | ከብት | 1. አለ፡ስንት------- | 0. የለም | |  | |
| 222 | ፈረስ/ አህያ/በቅሎ | 1. አለ፡ስንት------- | 0. የለም | |  | |
| 223 | ፍየል | 1. አለ፡ስንት------ | 0.የለም | |  | |
| 224 | በግ | 1. አለ፡ስንት------- | 0. የለም | |  | |
| 225 | ዶሮ | 1. አለ፡ስንት-------- | 0. የለም | |  | |
| 226 | የንብቀፎ | 1. አለ፡ስንት-------- | 0. የለም | |  | |

**ክፍል3-የስነባህሪጥያቄዎች (**መልሶቹንበመክበብወይምበባዶቦታውላይበመፃፍይግለÌ)

| 301 | በህይወትዘመንዎየአልኮልመጠጥሲጋራወይምጫት) ተጠቅመውያውቃሉ? | 1. አውቃለው | 0. አላውቅም |  |  |  |
| --- | --- | --- | --- | --- | --- | --- |
| 302 | ለጥያቄ 301. አዎከሆነመልስዎየትኛውንተጠቅመውያውቃሉ? | 1. የአልኮልመጠጥ(ቢራ፤ወይን፣አረቄ፣ጠላ፤ጠጅ) | | 2. ሲጋራማጭስ | 3. ጫት |  |
|  | ለጥያቄ 301 አላውቅምከሆነመልስዎወደጥያቄ 401 ይለፉ | | | |  |  |
| 303 | ባለፉት 3 ወራትውስጥከላይእጠቀመዋለውያሉትን (ሲጋራ/መጠጥ/ጫት)ለምንያህልግዜተጠቅመዋል? | | | | | |
|  | የአልኮልመጠጥ(ቢራ፤ወይን፣አረቄ፣ጠላ፤ጠጅ) | 1. ምንምግዜ | 2. አንዴወይምሁለቴ | 3. በወርአንዴ | 4.በየሳምንቱ | 5. በየቀኑ/ በየቀኑማለትይቻላል |
|  | ሲጋራ | 1. ምንምግዜ | 2. አንዴወይምሁለቴ | 3. በወርአንዴ | 4.በየሳምንቱ | 5. በየቀኑ/ በየቀኑማለትይቻላል |
|  | ጫት | 1. ምንምግዜ | 2. አንዴወይምሁለቴ | 3. በወርአንዴ | 4.በየሳምንቱ | 5. በየቀኑ/ በየቀኑማለትይቻላል |

**ክፍል 4-የማህበራዊእናየቤተሰብድጋፍመጠይቅ(**መልሶቹንበመክበብወይምበባዶቦታውላይበመፃፍይግለÌ)

| 401 | ድጋፍእያገኘህ/ኘሽነው? | 1= አዎ | 0= አላገኝም | ለጥያቄ 401 አላገኝምከሆነመልስዎወደጥያቄ 501 ይለፉ | |
| --- | --- | --- | --- | --- | --- |
| 402 | ምንአይነትድጋፍነውየምታገኚው/የምታገኘውከአንድበላይመልስመስጠትይቻላል፡፡ | 1= ስነ-ልቦናዊ | 2. የገንዘብድጋፍ | 3. አካላዊእንክብካቤ | 4. ሌላካለይግለጹ |
| 403 | ይህንንድጋፍየምታገኚው/ የምታገኘውከየትነውከአንድበላይመልስመስጠትይቻላል፡: | 1. ከጓደኞቼ  2. መንግስታዊካልሆኑተቋማት  3. ከቤተሰብ | 4. ህብረተሰብአቀፍከሆኑተቋማት | 5. ከመንግስታዊተቋምት  6. ከመስሪያቤት | 7. ከሀይማኖታዊተቋማት  8. ሌላካለይግለጹ |
| 404 | በአጠቃላይበምታገኘው/ኚውድጋፍምንያህልረክተሃል/ሻል | 1. አልረካሁም | 2. በመጠኑረክቻለሁ | 3. በጣምረክቻለሁ |  |
| 405 | የሚያስታምምህ/ሽሰውአለ? | | | 1. አዎ | 0. የለም |
| 406 | በችግር/በጭንቀትግዜጥሩምክርየሚሰጥህ/ሽሰውአለ? | | | 1. አዎ | 0. የለም |
| 407 | ወደሀኪምመሄድስትፈልግ/ጊየሚወስድህ/ሽሰውአለ? | | | 1. አዎ | 0. የለም |

| **ክፍል5 የመገለልናመድሎመጠይቅ** | | | | | |
| --- | --- | --- | --- | --- | --- |
|  | ባለፉት 6 ወርየተከሰቱ | በፍጹም | አንድጊዜ | ጥቂትጊዜ | ብዙጊዜ |
| 501 | ከማህበራዊስብሰባዎች/ስራዎችተግልዋል? |  |  |  |  |
| 502. | ከሀየማኖታዊቦታዎችተገለዋል? |  |  |  |  |
| 503. | ከበተሰብስራተገለዋል? |  |  |  |  |
| 504. | ስለርሶሰዎችመጥፎያስባሉ/ይታማሉወይ? |  |  |  |  |
| 505. | የቃላትትንኮሳሙከራደርሶብዎታል ? |  |  |  |  |
| 506. | ዐካላዊጥቃትደርሶብዎታል ? |  |  |  |  |
| 507. | ተሰድበውያውቃሉ? |  |  |  |  |
|  | **ድብርትን የተመለከተ** | | | | |
| 508ድ | ተበሳጫትተውያውቃሉ | - 1. አላውቅም 1. አዎ 2. ሁልጊዘማረፍአልቻልኩም 3. በጣምብስጩነኝ | | | |
| 509ድ | ተስፋምቁረጥደርሶብዎታል | 1. አላስብም 1.ያሳስበኛል 2. ተስፋየለኝም 3. በጣምደርሶብኛል | | | |
| 510ድ | የውድቀትስመት | 1. አላስብም 1. በመጠኑይሰማኛል 2. በጣምይሰማኛል 3. ሙሉበሙሉሰማኛል | | | |
| 511ድ | ራስንመጥላት | 0.አልጠላም 1. እጠላልሁ 2. በመጠኑእጠላልሁ 3. በጣምእጠላልሁ | | | |
| 512ድ | እንቅልፍማጣት | 1. አላጣም 1. ትዋትእነቃልሁ 2. 1-2 ሰአትቀድመእነቃልሁ 3. ሁለጊዘእንቃለሁ | | | |
| 513ድ | ከማህበረሰቡመገለል | 1. አልገለልም 1. በተቂቱእገለላሁ 2. ደርሶቢኛል 3. በታምደርሶብኛል | | | |

**አመሰግናለሁ፡፡**
