# Supplementary material for: Behavioral and psychosocial factors of quality of life among adult people living with HIV on Highly Active Antiretroviral Therapy, in public hospitals of Southwest Ethiopia
Source: PLOS Glob Public Health. 2022 Aug 12;2(8):e0000822. doi: 10.1371/journal.pgph.0000822 (PMC10022360; doi:10.1371/journal.pgph.0000822)
Supplement: Attachment — Submitted filename: Response to Reviewers..pdf [file pgph.0000822.s006.pdf]

Rebuttal letter

Response to reviewers

We thank the editor and reviewers for evaluating our manuscript. We provide line by line responses to comments raised. Please, follow our responses in a yellow mark to every comment/question in green mark. We copied the review decision from submission menu of the editorial manager. We used the same letter to write our responses. Plos, Global Health.

PGPH-D-21-00472
Behavioral and psychosocial factors of Quality of life among Adult people living with HIV on Highly Active Antiretroviral Therapy, in public hospitals of South West Ethiopia, 2018: A Case control study

PLOS Global Public Health

Dear Dr. Gesese,

Thank you for submitting your manuscript to PLOS Global Public Health. After careful consideration, we feel that it has merit but does not fully meet PLOS Global Public Health's publication criteria as it currently stands. Therefore, we invite you to submit a revised version of the manuscript that addresses the points raised during the review process.

Please submit your revised manuscript by Dec 02 2021 11:59PM. If you will need more time than this to complete your revisions, please reply to this message or contact the journal office at globalpubhealth@plos.org. When you're ready to submit your revision, log on to https://www.editorialmanager.com/pgph/ and select the 'Submissions Needing Revision' folder to locate your manuscript file.

Please include the following items when submitting your revised manuscript:

- A rebuttal letter that responds to each point raised by the editor and reviewer(s). You should upload this letter as a separate file labeled 'Response to Reviewers'.
- A marked-up copy of your manuscript that highlights changes made to the original version. You should upload this as a separate file labeled 'Revised Manuscript with Track Changes'.
- An unmarked version of your revised paper without tracked changes. You should upload this as a separate file labeled 'Manuscript'.

Guidelines for resubmitting your figure files are available below the reviewer comments at the end of this letter.

We look forward to receiving your revised manuscript.

Kind regards,

Olatunji O Adetokunboh, MD, PhD
Academic Editor
PLOS Global Public Health

Journal Requirements:

Editor Comments:

1. Please include additional information regarding the survey or questionnaire used in the study and ensure that you have provided sufficient details that others could replicate the analyses. For instance, if you developed a questionnaire as part of this study and it is not under a copyright more restrictive than CC-BY, please include a copy, in both the original language and English, as Supporting Information.

Responses: We thank you indeed for the advices on the revision. We have attached the questionnaires we used for the current survey. Please, check in the supporting information. This is labeled as S1-Appendix, S2-Appendix and S3-Appendix. We used local languages of Afan Oromo and Amharic. A translated and a pre-tested questionnaire were used on 15=5% of the respondents in one other hospital (Bedele General Hospital) than the study settings. Moreover, we used WHOQOL-HIV BREF tool that was validated and used else-where in Ethiopia.

2. In the online submission form, you indicated that "The data sets used and/or analyzed during the current study are available from the corresponding author of the research on reasonable request." All PLOS journals now require all data underlying the findings described in their manuscript to be freely available to other researchers, either 1. In a public repository, 2.Within the manuscript itself, or 3.Uploaded as supplementary information.
This policy applies to all data except where public deposition would breach compliance with the protocol approved by your research ethics board. If your data cannot be made publicly available for ethical or legal reasons (e.g., public availability would compromise patient privacy), please explain your reasons by return email and your exemption request will be escalated to the editor for approval. Your exemption request will be handled independently and will not hold up the peer review process, but will need to be resolved should your manuscript be accepted for publication. One of the Editorial team will then be in touch if there are any issues.

Responses: All relevant data are within the manuscript and its Supporting Information files.

3. State what role the funders took in the study. If the funders had no role in your study, please state: “The funders had no role in study design, data collection and analysis, decision to publish, or preparation of the manuscript.”

Responses: responded as “The funders had no role in study design, data collection and analysis, decision to publish, or preparation of the manuscript.” You can find in the updated manuscript.

Additional Editor Comments (if provided):

[Note: HTML markup is below. Please do not edit.]

Reviewers' comments:

Reviewer's Responses to Questions

Comments to the Author

1. Does this manuscript meet PLOS Global Public Health’s publication criteria? Is the manuscript technically sound, and do the data support the conclusions? The manuscript must describe methodologically and ethically rigorous research with conclusions that are appropriately drawn based on the data presented.

Reviewer #1: No

Reviewer #2: Partly

Reviewer #3: Partly

---

2. Has the statistical analysis been performed appropriately and rigorously?

Reviewer #1: No

Reviewer #2: No

Reviewer #3: Yes

---

3. Have the authors made all data underlying the findings in their manuscript fully available (please refer to the Data Availability Statement at the start of the manuscript PDF file)?

The PLOS Data policy requires authors to make all data underlying the findings described in their manuscript fully available without restriction, with rare exception. The data should be provided as part of the manuscript or its supporting information, or deposited to a public repository. For example, in addition to summary statistics, the data points behind means, medians and variance measures should be available. If there are restrictions on publicly sharing data—e.g. participant privacy or use of data from a third party—those must be specified.

Reviewer #1: No

Reviewer #2: No

Reviewer #3: Yes

---

4. Is the manuscript presented in an intelligible fashion and written in standard English?

PLOS Global Public Health does not copyedit accepted manuscripts, so the language in submitted articles must be clear, correct, and unambiguous. Any typographical or grammatical errors should be corrected at revision, so please note any specific errors here.

Reviewer #1: No

Reviewer #2: No

Reviewer #3: No

---

5. Review Comments to the Author

Please use the space provided to explain your answers to the questions above. You may also include additional comments for the author, including concerns about dual publication, research ethics, or publication ethics. (Please upload your review as an attachment if it exceeds 20,000 characters)

Reviewer #1: The authors submitted a manuscript reporting a study to determine the association of behavioral and psychosocial factors with the Quality of life among adult people living with HIV on Highly Active Antiretroviral Therapy (HAART) in selected public hospitals of South West Ethiopia. Whilst this is an important topic within the scope of this journal, the authors did not provide adequate scientific justifications for the study design, sample size calculation and stastical analysis. Also, the conclusions drawn from the study were not reflective of the study findings. More importantly, the manuscript was not well written in an acceptable standard English as it contains a lot of syntax errors and incomplete statements that made it difficult to understand or follow the lines of thoughts of the authors. I have highlighlighted some major concerns, while the minor comments were indicated within within the body of the manuscript:

Response: We thank you for your valuable comments on the paper. Considering an important topic within the scope of this journal, we have tried to provide adequate scientific justifications for the study design, sample size calculation and stastical analysis. Also, the conclusions drawn from the study were reflective of the study findings along with their respective recommendations. More importantly, the manuscript was tried to write in an acceptable Standard English with the considerable effort to edit. Therefore, we tried to respond to each question line by line.

1. Abstract: Given the title of this manuscript, it would be extremely important for the authors to include the name of the tool they used to assess the Quality of Life of the study participants.
How many of the participants were severely depressed among the cases and the controls? What tool was used to assess the study participants to arrive at this classification?

Response: Thank you very much for your insights and comments on the paper. In the abstract, we have now included the tool used to assess Quality of Life of the study participants. You can refer to line 21. Regarding participants with severe depression, in the earlier manuscript it was found in the result section but not in the abstract. It was to limit the word count. Now, you can find it in the result section of the abstract in line 28-29 and in line 239-240 of the result section under the heading psychosocial factors. Depression was measured using Beck depression

inventory (BDI-13score) by PCA considering its internal consistency and reliability with a (Crombach‘s Alpha 0.935) which is above o.7. It was measured using ordered scale from zero to three where the lower indicates minimal and the higher value indicated severe depression. You can find it in the operational definition under the supporting information files. It was not found for a similar reason to limit the word count in the abstract session.

2. Introduction: HIV/AIDS is no more a fatal illness as it has become a chronic manageable communicable disease, with advent of HAART, even in low-income countries Authors should check the follow recent publications: https://innovation.org/diseases/infectious/hiv-aids/hiv-aids-acute-fatal-disease-chronic-manageable-condition;

Response: we thank you for the reference provided. We have incorporated in the updated manuscript at lines 48-50 of the introduction session.

3.https://www.ncbi.nlm.nih.gov/pmc/articles/PMC3122586/

3.Authors should consider using a more recent data on national sero-prevalence of HIV in Ethiopia because the prevalence of 1.1% does not support the claim of Ethiopia being a country with high burden of HIV. Also, the national prevalence cited here was that of 2017. Data presenting the trends of the national prevalence of HIV in Ethiopia may be useful for this context: https://knoema.com/atlas/Ethiopia/HIV-prevalence

Response: Thank you for letting us uses the more recent data. It’s true that using more recent data can better transfer idea. While we were conducting this research during, we used that reference considered updated/recent data that time. We used USAIDS and PAPFAR, 2017 references. Per spectrum preliminary estimate by PEPFAR, January 2017, adult HIV prevalence in Ethiopia in 2016 was estimated to be 1.1%. There is substantial prevalence variation by region (6.6% in Gambella, 5.0% in Addis Ababa, and 0.7% in Southern Nations, Nationalities and Peoples’ (SNNPR) region). The HIV epidemic in Ethiopia is primarily associated with areas of urban concentration (5.1% in cities above 50 thousand compared to 3.1% in smaller cities and 0.6% in rural areas) and proximity to major transport corridors. Those living within five kilometers of a major road have HIV prevalence rates that are four-times higher than those who live further away. The two exceptions to this general pattern include Gambella region, a small and sparsely populated region that has the highest regional prevalence in Ethiopia (6.4%) and little distinction between urban and rural prevalence, and development schemes and seasonal migrant destinations that show elevated HIV-related risk behaviors despite not being close to urban areas or major roads. That showed great geographic area differences (with High prevalence in Gambella) with High in urban setting than rural. However, the HIV prevalence have shown decline from 2011 to 2016 EDHS reports. The study was taken place in an area where a high number of PLWHIV boarded with Gambella Region South West Ethiopia that accounts for the highest prevalence in Ethiopia. In line with this, a high number of travelers crossing the zone from Gambella region in which a large number of refugees and mining workers flee in to these study settings. Therefore, researchers considered these issues to undergo this research.

4. Authors should indicate whether this tool has been previously used in this setting or elsewhere in Ethiopia. Authors should also include the reliability and validity of the tool. Authors should indicate whether the English version of the instrument was used in the study, or a translated versions in local languages of the study participants were used. If yes, what were the reliability and validity indices of the translated versions of the instrument. Authors should check this link: https://ijmhs.biomedcentral.com/articles/10.1186/s13033-016-0062-x

Response: Thank you for us to see this reference. As you rose, we used a tool prepared in English that was translated in to local languages (Afan Oromo and Amharic) which is attached at the supporting information files. The tool has been used else-where in different studies of Ethiopia including in this study area.

5. This is a cumbersome definition of case and control definition for this study. WHOQoL-HIV BREF. What were the mean scores of WHOQoL-HIV BREF? How long were the PLWHIV enrolled in the HAART? Did the authors assess the quality of life of the PLWHIV before assigning recruiting them into either as a case or control in this study? These are difficult to understand from the authors' definition. Authors clarify these.

Response: Thank you! First of all, we used census to screening participants using the Overall Quality of Life Measured by using WHOQOL-HIV BIEF questionnaire that contains 31 questions distributed into 6 domains: physical, psychological, level of independence, social relationships, environmental domains and spirituality domains. The percentages mean score was calculated for each participant. Then, the mean score of percent mean scores was used as a cut-off to categorize the participants as poor (less than the mean score) and good (greater than or equal to the mean score) prior to the actual data collection tool was provided for data collection. This can be clearly seen in the questionnaire that is attached at the supporting information. Perhaps, this was challenging, however, we succeeded doing so. You can find it in data processing and analysis of tracked changes manuscript and the operational definitions under supporting information files.

6. Authors should support this sentence with appropriate references because ref 31 cited was not a case-control study. It was a cross-sectional hospital based study in Nigeria which has one of the highest burden of HIV in the world, after India and South-Africa. Using the prevalence obtained from a hospital-base cross-sectional study to determine the sample size of case-control study in a setting that is not similar to Nigeria, is not only inappropriate but also illogical.

Response: Thank you for the worthy comment. The sample size was calculated by using the double population proportion approach using statistical EPI info 7.1.1 software package by considering the percent of controls exposed of the psychosocial factor (having less social support) among controls (those with good quality of life) is 12% and among cases(those with poor quality of life) is 27.1% with AOR of 2.73 by considering the following parameters: 95% Confidence Level, 80% power, a case to control ratio of 1:3 accordingly, the final sample size, required was 300 (75 cases and 225 controls). The sample size was calculated by taking different factors from three different studies done [31-32, 34]. The final sample size, required was 300 (75 cases and 225 controls) by considering all objectives which gives more representative sample [32]. In this study, we used factors associated with quality of life

not the magnitude to calculate the sample size. We tried to calculate using a case control study, however, the absence of research in the field of study made it difficult and the sample that we found was small as compared to these studies. The other point you raised was about the differences in the prevalence of HIV. Finally, there are researches done elsewhere, using a cross-sectional study to calculate the sample size fir the case control study design. You can read these researches http://www.biomedcentral.com/1471-2431/14/91 and Yirsaw *et al. AIDS Res Ther (2020) 17:34.*

7. Was the questionnaire administered in English or in the local languages? What was the literacy level in the study population? If administered in local languages, was the questionnaire translated to the local languages through forward and backward translation? If not translated, how did the interviewers ensure consistency in administering the questionnaire to the study participants.

Response: thank you. As you rose, we used a tool prepared in English that was translated in to local languages (Afan Oromo and Amharic) then back translated in to English to see the consistency. This translated tool has been used to collect the data. As you rose about the literacy level in the study population, the majority of cases were less than grade 4 and controls were less than grade 8. As a result, we used the translated version using forward and backward translations to get valid data from participants. You can find it in lines from 163-165 of the tracked manuscript. Meanwhile, the tool has been used else-where in different studies in Ethiopia, including in the study area. It is attached at the supporting information as annex.

8. Authors should provide the justifications of $p<0.25$ for this study.

Response: Thank you indeed. It is well accepted comment. It is due to technical problem and on binary logistic regression analysis, a variable with P-value of ≤ 0.25 was used for candidate selection we accepted because candidates. It's corrected in the updated manuscript.

9. The references were not consistently cited. Authors should ensure the referencing styling was consistent; they should also provide missing accessed dated and correct URLs.

Response: Thank you indeed; the references are now consistently updated.

Reviewer #2: PGPH-D-21-00472

Thank you for the opportunity to review this manuscript which describes the behavioral and psychological factors affecting the quality of life of people living with HIV in Ethiopia. Despite the successful implementation of anti-retroviral therapy in many countries, PLHIV do face challenges that affect their quality of life, and it is important that the body of science in this area be expanded. Here are my comments to the authors.

In brief, the authors need to justify the need for this study clearly in the introduction section, the design used does not appear appropriate but if the authors want to keep it as a case-control design, then they must justify it based on principle and their study procedures. The results section requires significant improvement, this section must start

with a table showing the QoL assessments, so readers are able to see how the cases and controls scored on the assessments. Tables need to be numbered sequentially and the multivariable assessment need to be redone for BMI

Abstract

1. Please define your cases and controls clearly.

Response: Thank you indeed, the definition of cases and controls is given below. Case: Based on the WHOQOL-HIV BREF items, an individual patient living with HIV aged ≥15 years enrolled in to HAART care with poor quality of life (who scored below the mean value of WHOQOL-HIV BREF items as a cut of point) during census (the screening phase).

Control: An individual patient living with HIV and aged ≥15 years enrolled to HAART care with defined good quality of life (who scored above the mean value of WHOQOL-HIV BREF items as a cut of point) during the census (screening phase). What makes it vague was, census has been used to identify /screen cases and controls. You can find it in the lines from 21-24 of the abstract session.

2. Multivariate regression is different from multivariable regression. Did the authors assess risk factors for multiple outcomes and risk factors in the same model–that is what multivariate regression is

Response: Thank you! We used multivariate logistic regression analysis in which risk factors were run in one model regression. You can read in line 26 and in the methods section of the tracked manuscript..

3. Please give a brief description of the characteristics of the participants

Response: Thank you very much! We have added some descriptions in the abstract part despite we were not able to do because of limit to the word count. You can find in lines 27-29.

Introduction

1. Please copy edit and perhaps get an English editor the whole manuscript. There are many instances where an article "A" or "The" is missing at the start of sentences, where punctuations are missing, and where grammar needs to be revised.

Response: We thank you for advising us to make editions to the writing in English regarding this manuscript. We used "grammarly" an-online software to edit the spelling, grammar and language usage. Following that grammarly-online suggested there were word choice, grammar and punctuation issues, we approached PubSURE a research paper language editing service. Accordingly, we tried to respond by taking the comments by PubSure and left some that were illogical. Regarding use of professional editing, we don't afford to pay the required payment. Moreover, currently we have made changes in the manuscript in track changes and uploaded as *revised manuscript with track changes.* Meanwhile, we uploaded a clean copy of the edited manuscript with file name *manuscript* file

2. Please use scientific terms. The first sentence on page 4 needs to be specific about what the authors mean by "suffer"

"Despite different strategies provoked and tremendous efforts done to halt the spread and consequences of HIV/AIDS both at the global as well as national level, PLWHIV continued to suffer."

Response: Thank you very much! It is correct that we have modified the term. You can find it in line number 75 in the tacked changes.

3. The introduction introduces the concept of QL and HIV but does not show clearly where the gaps in current literature are and what gaps the current study intends to fulfill

Response: Thank you, the introduction introduces the concept of QL and HIV. We are hopeful that it clearly defines the gaps in the current literature and the current study intends to fulfill the gap. The study took place in an area where high numbers of PLWHIV were on ART. The Jimma zone is boarded by the Gambella Region, of Southwestern Ethiopia, which accounts for the highest HIV prevalence in Ethiopia as evidenced by a high number of travelers crossing the Gambella region known for its refugees and mining workers who put the town at high risk of contracting the disease [3, 39]. Given the benefits of measuring patients' treatment outcomes on HAART; there is a paucity of research on behavioral and psychosocial factors of quality of life in which, most of the studies were cross-sectional in nature that focused on clinical determinants [32-35]. With the demanding and significant benefit of monitoring and evaluation of patients' outcomes on HAART, this study aimed to identify behavioral and psychosocial factors affecting the quality of life among PLWHIV on HAART. We hope that you can find it in the introduction part of the paper in lines 97-106

Methods

1. There is no clear rationale why a case-control designed was used. Is poor QL a scarce outcome? It appears the authors samples by exposure and then assessed the outcome (using WHOQOL-HIV BREF).Or both at the same time – a classic cross sectional study, especially in view that there was no matching done

Response: Thank you very much indeed to see how much the method area deserves and needs critical understanding. Case control is used to identify risk factors. What we have gone so far is that, we used WHOQOL-HIV BREF tool to identify who of the respondents developed poor quality of life and the rest good quality of life. Overall, census has been done to come up with the main outcome of the study. Then after, we sent the cases (poor quality of life) and controls (with good quality of life) in to the actual data collection rooms. The main questionnaire was administered to identify risk factors associated with poor quality of life.

2. Please do not use the personal pronoun “I” as below “For the purpose of this study, i defined quality of life as personal evaluation of how things have been going for one self, and as how the individual‘s wellbeing may be impacted over time by a disease, a disability, or a disorder.”

Response: Thank you very much! It is admitted and corrected. You can see in the operational definition under the supporting information.

3. Please provide references for the definitions on page 7.

Response: Thank you very much! It’s ok. We have provided references for operational definitions. You may find it in the supporting information. You can see in the operational definition under the supporting information.

Results

1. By a response rate of 100%, do the authors mean that all the participants they approached agreed to take part? How many eligible individuals were there? A flow chart would be highly helpful

Response: all the participants they approached agreed to take part in this study. First, all cases were taken place with apparently random selection of controls. However, the controls with refusal were replaced by the next respondent. You can find it in line 222-223.

2. Please present the results of the WHOQOL-HIV BREF assessments.

Response: Regarding the results of the WHOQOL-HIV BREF assessments, these were not present because we used to screen study participants in order to incorporate them in to the main questionnaire. As a result, they were categorized in to cases and controls and not presented in the result session.

3. Please be consistent on decimal places. If you choose to use 1dp, then do so throughout, except for counts

Response: Thank you, we have accepted the comment and updated accordingly.

4. Tables should be numbered sequentially. Is there a reason to start with Table 2?

Response: Of course you are right; the reason was we did not present Table 1 that represents the sample size calculation although it was there. We have now corrected put sequentially in the updated manuscript.

5. In Table 2, what is the importance of distinguishing between?

Response: well accepted comment. Ok, we arranged accordingly.

6. Please put p-values in the Tables if you wish to then use those p-values as criteria for selection into multivariable models

Response: We have noted, however, we used a star to indicate the p-value instead of putting each p-value in the table form. You can find it at the end of table 5 of the included in the manuscript.

7. Table 5 – this is multivariable and not multivariate analysis

Response: we accepted that this is multivariable analysis. It's updated in the tracked changes. 8. Why is "obese" used as the base comparison for BMI categories – pleased use "normal"

Response: when we use referencing during analysis, we used the transformed value as overweight/obese. Therefore, we used this as reference for underweight respondents.

9. Please do not use bold for "significant results"

Response: Thank you, we have accepted and updated accordingly.

10. The model needs to be redone. Also, would be useful for the authors to submit the data underlying the study so reviewers (and readers) can run the models themselves. We are in a situation where Open Science has become more important than ever, and "data available from authors upon reasonable request" is no longer sufficient. However, this is my personal opinion and the authors should be guided by the journal policy

Response: Thank you, "data available from authors upon reasonable request" is a good idea. Of course, the data requires to be open access and we are happy to do so.

Reviewer #3: The authors conducted a case-control study to identify factors (data collected via a survey) associated with low perceived quality of life among people living with HIV in Ethiopia.

1. The manuscript requires revision to ensure correct grammar and syntax in English, as well as formatting (particularly in the tables).

Response: We thank you for advising us to make editions to the writing in English regarding this manuscript. We used "grammarly" online software to edit the spelling, grammar and language usage. Following that grammarly-online suggested there are word choice issues we approached Pub SURE a research paper language editing service. Accordingly, we tried to respond by taking the comments by Pub Sure and left some that were illogical. Regarding use of professional editing, we don't afford to pay the required payment. Moreover, currently we have made changes in the manuscript in track changes and uploaded as *revised manuscript with track changes.* Meanwhile, we uploaded a clean copy of the edited manuscript with file name *manuscript* file.

2. Participant recruitment is not clearly explained. A survey was conducted and survey data were used to define participants as cases and controls. But the final analysis data set matches exactly the sample size calculation, which does not seem to be representative of the overall distribution of QoL scores (1 case mean). How many total people were surveyed? How many survey participants who met the criteria for a case or control were then not included in the analysis? How was consecutive recruitment maintained in a 1:3 case-control ratio, when the definition of case and control is a mean survey score? A flow diagram detailed participant recruitment in the survey and inclusion in the analysis data set would be helpful.

Response: Thank you for the concern rose. Regarding participant recruitment, is was stated in the method part used the WHOQOL-HIV BREF tool to identify the respondents who developed poor quality of life and the rest good quality of life. Overall, census has been done to come up with the main outcome of the study. Then after, we sent the

cases (poor quality of life) and controls (with good quality of life) in to the actual data collection rooms. The main questionnaire was administered to identify risk factors associated with poor quality of life. After screening was done, all cases were enrolled consecutively, with random sampling used to apparently enroll controls. Since, we replaced controls all participants took place in the study.

3. Where did the estimated exposure to psychosocial factors in the sample size calculation come from?

Response: Thank you indeed. The sample size was calculated by using the double population proportion approach using statistical EPI info 7.1.1 software package by considering the percent of controls exposed of the psychosocial factor (having less social support) among controls (those with good quality of life) is 12% and among cases(those with poor quality of life) is 27.1% with AOR of 2.73 by considering the following parameters: 95% Confidence Level, 80% power, a case to control ratio of 1:3 accordingly, the final sample size, required was 300 (75 cases and 225 controls). The sample size was calculated by taking different factors from three different studies done [31-32, 34]. The final sample size, required was 300 (75 cases and 225 controls) by considering all objectives which gives more representative sample [32]. This reference used a study from Nigerian hospital. In this study, we used factors associated with quality of life not the magnitude to calculate the sample size. We tried to calculate using a case control study, however, the absence of research in the field of study made it difficult and the sample that we found was small as compared to these studies. The other point you raised was about the differences in the prevalence of HIV. Finally, there are researches done elsewhere, using a cross-sectional study to calculate the sample size fir the case control study design. You can read these researches http://www.biomedcentral.com/1471-2431/14/91 and Yirsaw *et al. AIDS Res Ther (2020) 17:34.*

4. Definitions could be included as supplementary material, rather than in the methods section.
Response:It is admitted and included in the supplementary material part/supporting information

5. Most of the findings from the multi-variate analysis (e.g. people experiencing food insecurity, disease stigma and depression have a low perceived quality of life) do not add anything new to the literature. The discussion confirms that these findings are not novel.

Response: Regarding the definitions, they are uploaded independently as operational definition in the supporting information files. In the discussion part, it stated that people experiencing food insecurity, disease stigma and depression have a low perceived quality of life. It’s obvious and noted in the present study, too. Although it seems redundant; it can inform responsible bodies to take different measures to come up with poor quality of life in areas like Ethiopia. It also can assure that these problems persisted despite of ART continued to be delivered.

---

6. PLOS authors have the option to publish the peer review history of their article (what does this mean?). If published, this will include your full peer review and any attached files.

Do you want your identity to be public for this peer review? If you choose “no”, your identity will remain anonymous but your review may still be made public.

For information about this choice, including consent withdrawal, please see our Privacy Policy.

Reviewer #1: **Yes:** Dr Muhammed Afolabi, London School of Hygiene & Tropical Medicine, UK

Reviewer #2: No

Reviewer #3: No

[NOTE: If reviewer comments were submitted as an attachment file, they will be attached to this email and accessible via the submission site. Please log into your account, locate the manuscript record, and check for the action link "View Attachments". If this link does not appear, there are no attachment files.]

While revising your submission, please upload your figure files to the Preflight Analysis and Conversion Engine (PACE) digital diagnostic tool, https://pacev2.apexcovantage.com/. PACE helps ensure that figures meet PLOS requirements. To use PACE, you must first register as a user. Registration is free. Then, login and navigate to the UPLOAD tab, where you will find detailed instructions on how to use the tool. If you encounter any issues or have any questions when using PACE, please email PLOS at figures@plos.org. Please note that Supporting Information files do not need this step.

Response: We have registered with PACE and all the tables and figures do fit with PLOS.. We downloaded from the PACE and included within the manuscript by copying the PDF using screen shot, and figure in PDF format. However, due to difficulty to make the TIF/ image file to an editable one, we used Microsoft excel in the recent updated manuscript to make it editable table. It is then included with in the manuscript. And the figure file is uploaded in the supporting information file separately but the legend is within the manuscript. You can find it in the supporting information for the figure as jpg form.

In compliance with data protection regulations, you may request that we remove your personal registration details at any time. (Remove my information/details). Please contact the publication office if you have any questions.

ReplyForward
