# Supplementary material for: Behavioral and psychosocial factors of quality of life among adult people living with HIV on Highly Active Antiretroviral Therapy, in public hospitals of Southwest Ethiopia
Source: PLOS Glob Public Health. 2022 Aug 12;2(8):e0000822. doi: 10.1371/journal.pgph.0000822 (PMC10022360; doi:10.1371/journal.pgph.0000822)
Supplement: Attachment — Submitted filename: Response to reviewers.docx [file pgph.0000822.s007.docx]

**Rebuttal letter**

**Response to reviewers**

We thank the editor and reviewers for evaluating our manuscript. We provide line by line responses to comments raised. Please, follow our responses in a yellow mark to every comment/question in green mark. We copied the review decision from submission menu of the editorial manager. We used the same letter to write our responses.

Plos, Global Health.

PGPH-D-21-00472

Manuscript: Behavioral and psychosocial 1 factors of Quality of life among Adult people living with HIV on Highly Active Antiretroviral Therapy, in public hospitals of South West Ethiopia, 2018: A Case-control study

**Reviewer #5.**

Summary: This manuscript works to define behavioral and psychological factors that most affect the quality of life of PLHIV in the Gambella region of Ethiopia. This region is unique both in the rate of HIV and rural and urban distribution. The manuscript seeks to establish challenges that PLHIV face despite the availability of HAART. Initial review of this manuscript was focused on the improvement of statistical analysis and model selection. Reviewers were concerted that the definitions of case and control groups were unclear and were confused on how participants were enrolled in the study.

**Major concerns:**

1. Although an obvious effort has been made to improve the standard English of the manuscript, there are still many areas of the manuscript that could be improved in grammar and clarity. Attention needs to be paid to a consistency in acronym definitions and usage as there are many instances where an acronym is defined twice or not used after definition. Furthermore, consistent verb tense should be ensured.

**Response**

Thank you indeed! It’s well accepted comment. We have gone through the manuscript to improve the standard language of the manuscript. We improved consistency in acronym definitions and usage.

2. In your introduction you speak of how important being near a major roadway impacts the likelihood of being a PLHIV. That being said, the majority of cases are from rural areas and the majority of controls are from urban areas. Does this introduce bias?

**Response**

As it was stated in the introduction session of this session, the study took place in an area where high numbers of PLWHIV were on ART, and methods section from lines 112-115 Addis Ababa, Amhara, Oromia and Former Southern Nations Nationalities People Region (SNNPR) account for 93% of total PLWHIV in the country, and with a high urban prevalence (60%) in cities, and towns. It is also found near Gambella region, a region that accounts for the highest prevalence rate (6.5%) of HIV from Ethiopia [3]. The study took place in public hospitals found in the zone and found in different rural towns. This might be the reason that cases are from rural areas and the majority of controls are from urban areas. At the same time, bias might have been encountered though efforts had been used to control it. This has been stated in the limitation session.

3. Furthermore, do you think the incident of refugees and worker fleeing impacts the rate of psychological distress. I think a discussion of the Gambella region and the incidence of refugees in the population should be discussed. In the response to reviewers, the authors emphasize the uniqueness of the Gambella region of Ethiopia. This discussion would be improved as it adds relevance to the manuscript.

**Response**

Thank you for sharing an interesting comment. Its well accepted that PLWHIV, might have been exposed to psychological distress associated high turnover of individuals, and workers from refugees, and mining workers of Gambella region with a high prevalence of HIV and one of the hot spot areas of disease in the country. As a result of this, PLWHIV might have had used substances to cope up with the adverse effects of distress which in turn, affects their QOL. This portion is included in the discussion session from lines 291-295.

4. The authors did not address the original reviewers concerns with statistics. Although more detail was provided, no changes were made to the analysis.

**Response**

Thank you indeed. It’s very important comment and we have accepted it that we used bivariate and multivariable logistic regression analysis.

**Minor concerns:**

Ln 13/14: if you are going to use HAART and PLWHIV in the abstract it would be worthwhile to define them here.

**Response**

Thank you! It’s well accepted comment. We have updated it.

Ln 18: lowercase Q in “quality of life’. This error is repeated throughout the manuscript.

**Response**

Thank you! It’s well accepted comment. We have tried to update it.

Ln 21: delete / after 10

**Response**

Thank you! It’s well accepted comment. Rather, we have modified it.

Line 23: Apparently random? What does this mean?

**Response**

Thank you! It’s well accepted comment. We have removed and updated.

Ln 43:“HIV is”

**Response**

Thank you! It’s well accepted comment. We have updated it.

Ln 49: “chronic, manageable”

**Response**

Thank you! It’s well accepted comment. We have updated it.

Ln 52: The sentence that starts “It helps…” is not clear and may not be necessary for the understanding of the topic at hand

**Response**

Thank you! It’s well accepted comment. We have updated it.

Ln 54: “provides maximal” … “restores and/or preserves”

**Response**

Thank you! It’s well accepted comment. We have updated it. You can find in Line 56.

Ln 57-59: hard to understand, not sure what the sentence is trying to portray

**Response**

Thank you! It’s well accepted comment. We have updated it in the updated manuscript.

Ln 60: “PLWHIV, which”

**Response**

Thank you! It’s well accepted comment. We have updated it.

Ln 62: Define QOL at the first use, many lines above

**Response**

Thank you! It’s well accepted comment. We have updated it.

Ln 65 “such as”

**Response**

Thank you! It’s well accepted comment. We have updated it

Ln 73: I think you may have meant to write ‘invoked’; however, I think you may want to use a different work as invoked isn’t quite what you’re trying to portray

**Response**

Thank you! It’s well accepted comment. We have updated it in Line 75.

Ln 74: ‘level’

**Response**

Thank you! It’s well accepted comment. We have updated it.

Ln 78: you’ve already defined QOL above

**Response**

Thank you! It’s well accepted comment. Although we have defined QOL in the earlier one, it was defined different by different organization which is aimed at observing from different dynamics.

Ln 78: “In a 2015 World Health Organization report,…”

**Response**

Thank you! It’s well accepted comment. We have updated it and find it in line 77.

Ln 87: “It has been associated, in a complex way, with impoverished…”

**Response**

Thank you! It’s well accepted comment. We have updated.

Ln 110: Zone is capitalized in the line above but not here. Keep consistent if Jimma Zone is a proper noun

**Response**

Thank you! It’s well accepted comment. We have updated across the paper.

Ln 125: as items is plural, remove “the” before WHO

**Response**

Thank you! It’s well accepted comment. We have updated it.

Ln 132: “five indicates”

**Response**

Thank you! It’s well accepted comment. We have updated it.

Ln 137: “cut off”

**Response**

Thank you! It’s well accepted comment. We have updated it.

Ln 142: This is a very long sentence and very difficult to understand; however, this sentence is integral to understanding study design.

**Response**

Thank you! It’s well accepted comment. We have updated it.

Ln 147: which factors were considered from these studies?

**Response**

Thank you! We have reported these variables. We used having less and moderate social support, lower income from other studies. You can find it in the table 1.

Table 1: What’s the difference between the first “Psychological (having less social support)” and the second?

**Response**

Thank you! It’s psychosocial is the variable and having less social support if the category which we used to calculate the sample size.

Table 2: Where is table 1? The table titles do not match in text tables

**Response**

Thank you! It’s well accepted comment. It’s in the supplementary file.

Ln 165: how was the validity of the tool ensured?

**Response**

Thank you! It’s well accepted comment. We have updated and put the value in Lines 162-163.

Cronbach**’s** alpha of 0.803 was used to test for internal consistency and reliability of the overall QOL. We used a tool which was used by other researchers in Ethiopia, including the study area.

Ln 170: the case and control structure has been explained many times. I don’t think this amount of redundancy is unnecessary

**Response**

Thank you! Well accepted comment. We used it to clearly indicate each procedure conducted in this study.

Ln 179: I think you mean mentioned?

**Response**

Thank you! It’s to mean mentored. In line-177.

Ln 206: instead of “kept” consider using “ensured” for more clarity

**Response**

Thank you! It’s updated.

Ln 207: “faced”

**Response**

Thank you! It’s updated. Line 204.

Ln 208: “supported”

**Response**

Thank you! Updated in line 205 in the updated.

Ln 208: what is an example of a problem incurred by data collectors?

**Response**

Thank you! Sometimes data collects may encounter, problems when some participants misconduct during the interview, difficulty in understanding of questions፣ inconsistencies in collected data, and blank/ incomplete data.

Ln 213: Health Department should be capitalized as it is a proper noun, line 210.

**Response**

Thank you! It is updated.

Ln 215: “facility”

**Response**

Thank you! In line 212.

Ln 218: Choose between informants and participants and stay consistent throughout

**Response**

Thank you! We have tried to use participants to make the consistency of words.

Ln 235: Regarding the comment on table 2, do you think the fact that cases experienced less psychosocial support is due to the culture of rural lifestyle?

**Response**

Thank you! Well accepted comment. It might be due to the fact that cases experienced less psychosocial support is due to the culture of rural lifestyle.

Table 5: It is interesting that 61% of controls claimed to receive support but the majority of specific support questions resulted in less than 20% receiving support

**Response**

Thank you! Although the majority of controls claim to receive support, resulted in less than 20% receiving support

Ln 280: associated with what

**Response**

It’s updated.

Ln 320: “more” not “most”

**Response**

It’s updated in line 322.

Ln 335: should add PLWA here as it is used throughout the manuscript.

**Response**

Thank you! We used PLWHIV throughout the manuscript.

**Reviewer # 2**

Review - PGPH-D-21-00472

Thank you to the authors for the revised manuscript. Although the authors have addressed some of the concerns I raised earlier, this paper is still not ready for publication. I have detailed some concerns below:

1. Case control design samples participants using the outcome, usually this is in a situation where the outcome is scarce. However, one could use a case control when the outcome is not scarce – as in this scenario – if they so choose and then carry out the appropriate analysis. The distinguishing feature of a case control study is the directionality – from outcome (measured first) to the exposure (measured after outcome ascertainment). This is not the case in this study. The authors carried out a cross sectional study and then simply divided their participants into those with the outcome, which they defined as poor quality of life (who scored below the mean value of WHOQOL-HIV BREF items as a cut of point)(this definition is also problematic, but it’s a separate point, which again should be addressed). The authors’ response is not adequate – there is no justification for turning a cross sectional study such as this one into a case control study – simply because the authors have individuals with an outcome. What matters is the directionality of the study in this study both outcome and exposures were assessed in a cross-sectional manner.

**Response:**

Thank you for sharing your experiences and comments! As we have noticed earlier, as you have tried to raise, we tried to find the outcome (measured first) to the exposure (measured after outcome ascertainment). During such instance, we used to screen to identify case participants with poor quality of life. This has happened earlier before the actual data collection (on exposure variables has taken place). Once, we selected and identified the outcome, we sent the participants in to the actual data collection rooms, placed in separate areas. The identification of cases was kept confidential for data collectors and it was marked on the chart for identification. After that, data on exposure variables of the main questionnaire was conducted. What was challenging for us is the issue of clearly identifying cases and controls with such a vast number of questions and calculates/ identifies which might expose for misclassification/selection bias though efforts have been made to reduce it. We have explained this issue in the limitation session. Since it was also a multi centre study, we were exhausted in doing so.

2. The authors need to explicitly describe the study procedures early in the methods section so the reader is able to understand exactly what happened in this study. What was the sequence of events once a participant was recruited into the study? Again, the authors are advised to present a flow chart so readers may understand the recruitment and inclusion into the study. For example, when was “screening” done? When were the risk factors assessed?

**Response:**

Data were collected by two ART nurses working from each hospital and who had prior work experience in ART clinics. A face-to-face interview using an interviewer-administered structured questionnaire was conducted. The sequence of events is stated below. First, a census/ screening of patients who were on ART care at each hospital was conducted using the WHOQOL-HIV BREF instrument. This tool was used to screen participants as cases with poor Quality of life and controls with good Quality of life by considering the mean score as cut off point. After screening cases and controls, participants were sent to two data collection rooms. Then, the main information on exposure variables (socio-demographic and economic, behavioral, and psychosocial characters) collected. All cases were enrolled consecutively. For each case, three controls were enrolled using a simple random sampling technique in which non respondents were replaced. A flow diagram is attached as a fig file in the manuscript. One supervisor was assigned to each site to oversee the process. The principal investigator supervised every aspect of the data collection process along with supervisors and the ART nurses mentored shouldering the regular data collection task. The filled questionnaire was gathered by the principal investigator and/or the supervisor on a daily basis.

3. The definitions of poor quality of life (who scored below the mean value of WHOQOL-HIV BREF items as a cut of point), or good quality of life (who scored above the mean value of WHOQOL140HIV BREF items as a cut of the point) as I stated above, is inadequate. Simply scoring below or above a sample mean does define a good/poor outcome as this is neither valid internally nor valid externally. Within a sample, the mean simply tells us the average WHOQOL – it cannot be used to define poor/good outcomes. If the authors want to use sample statistics, in the absence of valid cut-offs of the WHOQOL scores, it may be advisable to use the extremes, for example the lower 25^th^ percentile WHOQOL score on these scores although even that lacks external validity.

**Response**

Thank you for your comment! If the authors want to use sample statistics, in the absence of valid cut-offs of the WHOQOL scores, it may be advisable to use the extremes, for example the lower 25^th^ percentile WHOQOL score on these scores although even that lacks external validity. However, we have employed using mean.

4. Where is Table 1?

**Response**

It is in the supplementary file leveled as table 1.

5. Where is Fig 1?

**Response**

It is uploaded as fig 1 file.

6. Where is Table 3?

**Response**

It is in ln 235.

7. Tables 2 on line 151 is unnecessary and should put in supplementary materials

**Response**

It is renamed as table 1. And, it is found in the main manuscript file.

8. There are two Table2’s – please rename one of them

**Response**

It is renamed, table 1 and 2 are now correctly ordered and found in the main table and sequentially ordered.

9. The statement below implies that some individuals were chosen to be cases (all) and then controls (unmatched) selected from the remaining pool of participants in a ratio of 1:3.

“First, we used the census to screen participants using the WHOQOL-HIV BREF instrument by considering the mean score as cut off point for poor quality of life (cases) and good quality of life (controls). Then, all cases were enrolled consecutively with three controls using a simple random sampling technique. After screening cases and controls, respondents were sent to two data collection rooms, and face to face interviews were conducted to collect information.”

What criteria was used to select controls? If the approach implied here – that some individuals with QOL above the mean were chosen and others excluded, there is a real risk of selection bias – how did the authors ensure that the controls they chose were similar to those they excluded? How many were excluded? Again, this takes us back to the study design – that the design is a cross-sectional study and should be reported and analyzed as such.

**Response**

We have included the inclusion criteria of cases and controls.

Adult People living with HIV aged ≥15 years and started ART treatment and at all stages of HIV disease screened with poor QOL for cases and with good QOL for controls. However, controls with incomplete data and who were not willing to participate were replaced by other participants. Of course, almost all approached participants were included except few which were replaced. You can find it in the methods section. Regarding the risk of selection bias, though efforts have been tried to limit or control, this issue is reported in the limitation of the study.

10. The authors insist that they carried out a multivariate analysis. This is not possible, as they only had one outcome in their analysis– this analysis should be reported as, multiple variable logistic regression, and not “multivariate”.

**Response**

Thank you indeed! We had one outcome with multiple factors. Its well accepted comment and we have amended in the updated manuscript.

11. The authors used a statistical cut-off of p less than or equal to 0.25 to decide which variables went into their multiple variable logistic regression. This is not a robust method and risks some chance findings in these analyses. Rather, the decision to include variables should be based on other robust methods such as directed acyclic graphs or a review of literature and mechanisms between exposure and outcome.

**Response**

A P-value of <=0.25 in the bivariate analysis and those variables which frequently showed significant association in the previous studies, regardless of the p-value, were included in to multivariable model. You can find it in lines 191-193.

12. The authors have chosen to denote statistical significance using an asterik. This is not very useful as it assumes that some cut-off (such as 0.05) has scientific merit – a debatable point. In any case, it may be worthwhile to present the actual p-values themselves and let the reader have a clear idea of the degree of evidence against each of the null hypotheses being tested. On a positive note, the authors have.

**Response**

To reduce the confusion/debate on asterisk, we have presented the 95% CIs, and p-values at different levels which we have updated in the uploaded manuscript. You can find it in table 5.

13. The analysis in Table 5 needs to be redone and should include known risk factors for poor quality such as gender, age, socio-economic status variables and alcohol use

**Response**

Thank you for your comment. We have included those variables in the analysis. However, they we not fitted in to the final model. This has been elaborated in the analysis session of the updated manuscript.

14. An interesting finding from this study is that participants who chewed khat were more likely to have a poor quality of life. What are the mechanisms involved?

**Response**

In settings where khat use is common, developing targeted behavior-change interventions as the health priority should be included in the comprehensive care package for PLWHIV. This can also improve life skills associated with behavioral factors. This is presented both in the manuscript and conclusion part. Further see lines number 326-329.
