# Supplementary material for: Behavioral and psychosocial factors of quality of life among adult people living with HIV on Highly Active Antiretroviral Therapy, in public hospitals of Southwest Ethiopia
Source: PLOS Glob Public Health. 2022 Aug 12;2(8):e0000822. doi: 10.1371/journal.pgph.0000822 (PMC10022360; doi:10.1371/journal.pgph.0000822)
Supplement: Attachment — Submitted filename: Response to reviewers.docx [file pgph.0000822.s008.docx]

**Rebuttal letter**

**Response to reviewers**

We thank the editor and reviewers and editors for evaluating our manuscript. We provide line by line responses to comments raised. Please, follow our responses in a yellow mark to every comment/question in green mark. We copied the review decision from submission menu of the editorial manager. We used the same letter to write our responses. Plos, Global Health.

PGPH-D-21-00472
Behavioral and psychosocial factors of Quality of life among Adult people living with HIV on Highly Active Antiretroviral Therapy, in public hospitals of South West Ethiopia, 2018: A Case control study

CC: [olatunji@sun.ac.za](mailto:olatunji@sun.ac.za), [adetok_sic@yahoo.com](mailto:adetok_sic@yahoo.com)
Thank you for submitting your manuscript to PLOS Global Public Health. After careful consideration, we feel that it has merit but does not fully meet PLOS Global Public Health’s publication criteria as it currently stands. Therefore, we invite you to submit a revised version of the manuscript that addresses the points raised during the review process.
Response

Thank you the editor for your careful consideration having a merit on the article. We have made a rigorous effort to make the paper worth enough to be considered for publication and bring about a change to the scholars.

Please submit your revised manuscript by Jul 03 2022 11:59PM. If you will need more time than this to complete your revisions, please reply to this message or contact the journal office at [globalpubhealth@plos.org](mailto:globalpubhealth@plos.org). When you're ready to submit your revision, log on to <https://www.editorialmanager.com/pgph/> and select the 'Submissions Needing Revision' folder to locate your manuscript file.

Please include the following items when submitting your revised manuscript:

- A rebuttal letter that responds to each point raised by the editor and reviewer(s). You should upload this letter as a separate file labeled 'Response to Reviewers'.
- A marked-up copy of your manuscript that highlights changes made to the original version. You should upload this as a separate file labeled 'Revised Manuscript with Track Changes'.
- An unmarked version of your revised paper without tracked changes. You should upload this as a separate file labeled 'Manuscript'.

Guidelines for resubmitting your figure files are available below the reviewer comments at the end of this letter.

We look forward to receiving your revised manuscript.

Kind regards,

Olatunji O Adetokunboh, MD, PhD
Academic Editor
PLOS Global Public Health

Journal Requirements:

Additional Editor Comments (if provided):
[Note: HTML markup is below. Please do not edit.]
Reviewers' comments:
Reviewer's Responses to Questions

**Response to Reviewer #2**

**Comments to the Author**

1. If the authors have adequately addressed your comments raised in a previous round of review and you feel that this manuscript is now acceptable for publication, you may indicate that here to bypass the “Comments to the Author” section, enter your conflict of interest statement in the “Confidential to Editor” section, and submit your "Accept" recommendation.

Reviewer #2: All comments have been addressed

2. Does this manuscript meet PLOS Global Public Health’s [publication criteria](https://journals.plos.org/globalpublichealth/s/criteria-for-publication)? Is the manuscript technically sound, and do the data support the conclusions? The manuscript must describe methodologically and ethically rigorous research with conclusions that are appropriately drawn based on the data presented.

Reviewer #2: No

Response

Since we have updated the paper including the method part particularly the study design and analysis we are hopeful that our paper is upgraded that the conclusion is supported by the data presented from this research.

3. Has the statistical analysis been performed appropriately and rigorously?

 Reviewer #2: No

Response

The statistical analysis has been performed appropriately and rigorously in the updated manuscript. We have used the WHOQOL-HIV BRIEF tool coded properly. You can see in this section in the updated manuscript.

 4. Have the authors made all data underlying the findings in their manuscript fully available (please refer to the Data Availability Statement at the start of the manuscript PDF file)?
The [PLOS Data policy](https://journals.plos.org/globalpublichealth/s/data-availability) requires authors to make all data underlying the findings described in their manuscript fully available without restriction, with rare exception. The data should be provided as part of the manuscript or its supporting information, or deposited to a public repository. For example, in addition to summary statistics, the data points behind means, medians and variance measures should be available. If there are restrictions on publicly sharing data—e.g. participant privacy or use of data from a third party—those must be specified.

 Reviewer #2: No

Response

Thank you for your comment. We have updated in the manuscript and in the system too. We can provide the research data up on reasonable request from the correspondent author.

 5. Is the manuscript presented in an intelligible fashion and written in standard English?

PLOS Global Public Health does not copyedit accepted manuscripts, so the language in submitted articles must be clear, correct, and unambiguous. Any typographical or grammatical errors should be corrected at revision, so please note any specific errors here.

 Reviewer #2: No

Response

Thank you for your comment. We have tried to edit the language and grammatical errors.

 6. Review Comments to the Author

Please use the space provided to explain your answers to the questions above. You may also include additional comments for the author, including concerns about dual publication, research ethics, or publication ethics. (Please upload your review as an attachment if it exceeds 20,000 characters)

**Over all responses to comments**

Reviewer #2: Dear Editor, the authors have responded well to some of the issues I raised earlier. However, the design and analysis of this paper is still not adequate, and the paper is not suitable for publication.

Minor issues

This paper requires proof reading by an English expert as there are many grammatical and language errors.

**Major issues**

Study design

The authors response is noted. I am still not convinced that the case control design is the correct design for this study. This is worsened by an inadequate case definition using a cut-off based on the mean in their sample. This is not a correct definition of “poor” quality of life.

Response

We really thank you very much on the major issues raised by the reviewer. We have tried to do the analysis and amended according to the comments given. A cross sectional study design was conducted in public hospitals of Jimma Zone, Southwest Ethiopia from March 10 to April 30/2018. As a result, we have done the analysis based on the standard coding formats. You see the updated manuscript files.

1. Can the authors please confirm that they coded the WHOQOL-HIV BRIEF correctly as in the WHO manual <https://apps.who.int/iris/rest/bitstreams/109786/retrieve>

Response

Since we have re analyzed the data, we have coded correctly using the above WHO manual.
2. Where are the data for the WHOQOL-HIV BRIEF and the other data in the study?

Response

Regarding data on WHOQOL-HIV BRIEF, we have presented the results for each and overall domain scores. See **table 4** in the updated manuscript files?
3. The WHOQOL-HIV BRIEF does not provide cut-offs, which could have been useful for the authors to use to define their cases and controls. In the absence of validated cut-offs, the authors are advised to analyse the QoL as a continuous outcome, in a cross-sectional design rather than a case control. An example of this approach is here <https://www.tandfonline.com/doi/full/10.1080/09540121.2016.1146209>

If the authors choose to continue with a cross sectional design, the best way to do so is to define their cases using the extremes (25th percentile or lower) as these are individuals who most likely have significant lower QoL than the others in the sample.

Response

Thank you indeed for your comments. Although QOL is a continuous outcome, we have analyzed and updated it; Domain scores in the WHOQOL-HIV were scaled in positive direction with higher score denoting good quality of life. Negative questions like pain and discomfort were recorded so that higher scores reflected better QOL. Mean scores of the items within each domain were used to calculate the domain score. Mean scores were then multiplied by 4 in order to make domain score comparable with the scores used in WHOQOL-100 (15). By taking the mean scores of each domain and the overall QoL as a cutoff point, QOL was dichotomized as poor or good. Individuals who scored below the mean were classified as having poor quality of life on each of the six domains. You can access this in the methods section of data analysis part from lines 155-162. In addition we have used references published elsewhere. You can see these references (22, 40,42)

**Minor issues**

4. This paper requires proof reading by an English expert as there are many grammatical and language errors

Response

Thank you! We have tried to edit grammatical and language issues.

 7. PLOS authors have the option to publish the peer review history of their article ([what does this mean?](https://journals.plos.org/globalpublichealth/s/editorial-and-peer-review-process#loc-peer-review-history)). If published, this will include your full peer review and any attached files.
**Do you want your identity to be public for this peer review?** If you choose “no”, your identity will remain anonymous but your review may still be made public.

For information about this choice, including consent withdrawal, please see our [Privacy Policy](https://www.plos.org/privacy-policy).

Reviewer #2: **Yes:** Tawanda Chivese

**Response to Reviewer #4**

**Comments to the Author**

1. If the authors have adequately addressed your comments raised in a previous round of review and you feel that this manuscript is now acceptable for publication, you may indicate that here to bypass the “Comments to the Author” section, enter your conflict of interest statement in the “Confidential to Editor” section, and submit your "Accept" recommendation.

Reviewer #4: All comments have been addressed

 2. Does this manuscript meet PLOS Global Public Health’s [publication criteria](https://journals.plos.org/globalpublichealth/s/criteria-for-publication)? Is the manuscript technically sound, and do the data support the conclusions? The manuscript must describe methodologically and ethically rigorous research with conclusions that are appropriately drawn based on the data presented.

 Reviewer #4: Yes

 3. Has the statistical analysis been performed appropriately and rigorously?

 Reviewer #4: Yes

4. Have the authors made all data underlying the findings in their manuscript fully available (please refer to the Data Availability Statement at the start of the manuscript PDF file)?

The [PLOS Data policy](https://journals.plos.org/globalpublichealth/s/data-availability) requires authors to make all data underlying the findings described in their manuscript fully available without restriction, with rare exception. The data should be provided as part of the manuscript or its supporting information, or deposited to a public repository. For example, in addition to summary statistics, the data points behind means, medians and variance measures should be available. If there are restrictions on publicly sharing data—e.g. participant privacy or use of data from a third party—those must be specified.

Reviewer #4: Yes

5. Is the manuscript presented in an intelligible fashion and written in standard English?

PLOS Global Public Health does not copyedit accepted manuscripts, so the language in submitted articles must be clear, correct, and unambiguous. Any typographical or grammatical errors should be corrected at revision, so please note any specific errors here.

Reviewer #4: No

Response

Thank you for your comment! We have tried to edit these language and grammar errors in the updated manuscript.

 6. Review Comments to the Author

Please use the space provided to explain your answers to the questions above. You may also include additional comments for the author, including concerns about dual publication, research ethics, or publication ethics. (Please upload your review as an attachment if it exceeds 20,000 characters)

Reviewer #4: Thank you for addressing all necessary comments.

 7. PLOS authors have the option to publish the peer review history of their article ([what does this mean?](https://journals.plos.org/globalpublichealth/s/editorial-and-peer-review-process#loc-peer-review-history)). If published, this will include your full peer review and any attached files.

**Do you want your identity to be public for this peer review?** If you choose “no”, your identity will remain anonymous but your review may still be made public.

For information about this choice, including consent withdrawal, please see our [Privacy Policy](https://www.plos.org/privacy-policy).

Reviewer #4: No

[NOTE: If reviewer comments were submitted as an attachment file, they will be attached to this email and accessible via the submission site. Please log into your account, locate the manuscript record, and check for the action link "View Attachments". If this link does not appear, there are no attachment files.]

While revising your submission, please upload your figure files to the Preflight Analysis and Conversion Engine (PACE) digital diagnostic tool, <https://pacev2.apexcovantage.com/>. PACE helps ensure that figures meet PLOS requirements. To use PACE, you must first register as a user. Registration is free. Then, login and navigate to the UPLOAD tab, where you will find detailed instructions on how to use the tool. If you encounter any issues or have any questions when using PACE, please email PLOS at [figures@plos.org](mailto:figures@plos.org). Please note that Supporting Information files do not need this step.

*In compliance with data protection regulations, you may request that we remove your personal registration details at any time.* [*(Remove my information/details)*](https://www.editorialmanager.com/pgph/login.asp?a=r)*. Please contact the publication office if you have any questions.*

|  | ReplyForward |
| --- | --- |
